# Association of Language Barriers With Perioperative and Surgical Outcomes: A Systematic Review

**DOI:** 10.1001/jamanetworkopen.2023.22743

**Published:** 2023-07-11

**Authors:** Hyundeok Joo, Alicia Fernández, Elizabeth C. Wick, Gala Moreno Lepe, Solmaz P. Manuel

**Affiliations:** 1 Department of Epidemiology and Biostatistics, University of California, San Francisco, San Francisco; 2 Department of Preventive Medicine, Seoul National University College of Medicine, Seoul, Republic of Korea; 3 Department of Medicine, University of California San Francisco School of Medicine, San Francisco; 4 UCSF Center for Vulnerable Populations, Zuckerberg San Francisco General Hospital, San Francisco, California; 5 Department of Surgery, University of California San Francisco School of Medicine, San Francisco; 6 University of California San Francisco School of Medicine, San Francisco; 7 Now with Department of Internal Medicine, Brigham and Women’s Hospital, Boston, Massachusetts; 8 Department of Anesthesia and Perioperative Care, University of California San Francisco School of Medicine, San Francisco

## Abstract

**Importance:**

English language proficiency has been reported to correlate with disparities in health outcomes. Therefore, it is important to identify and describe the association of language barriers with perioperative care and surgical outcomes to inform efforts aimed at reducing health care disparities.

**Objective:**

To examine whether limited English proficiency compared with English proficiency in adult patients is associated with differences in perioperative care and surgical outcomes.

**Evidence Review:**

A systematic review was conducted in MEDLINE, Embase, Web of Science, Sociological Abstracts, and CINAHL of all English-language publications from database inception to December 7, 2022. Searches included Medical Subject Headings terms related to language barriers, perioperative or surgical care, and perioperative outcomes. Studies that investigated adults in perioperative settings and involved quantitative data comparing cohorts with limited English proficiency and English proficiency were included. The quality of studies was evaluated using the Newcastle-Ottawa Scale. Because of heterogeneity in analysis and reported outcomes, data were not pooled for quantitative analysis. Results are reported in accordance with the Preferred Reporting Items for Systematic Reviews and Meta-Analysis Protocols guideline.

**Findings:**

Of 2230 unique records identified, 29 were eligible for inclusion (281 266 total patients; mean [SD] age, 57.2 [10.0] years; 121 772 [43.3%] male and 159 240 [56.6%] female). Included studies were observational cohort studies, except for a single cross-sectional study. Median cohort size was 1763 (IQR, 266-7402), with a median limited English proficiency cohort size of 179 (IQR, 51-671). Six studies explored access to surgery, 4 assessed delays in surgical care, 14 assessed surgical admission length of stay, 4 assessed discharge disposition, 10 assessed mortality, 5 assessed postoperative complications, 9 assessed unplanned readmissions, 2 assessed pain management, and 3 assessed functional outcomes. Surgical patients with limited English proficiency were more likely to experience reduced access in 4 of 6 studies, delays in obtaining care in 3 of 4 studies, longer surgical admission length of stay in 6 of 14 studies, and more likely discharge to a skilled facility than patients with English proficiency in 3 of 4 studies. Some additional differences in associations were found between patients with limited English proficiency who spoke Spanish vs other languages. Mortality, postoperative complications, and unplanned readmissions had fewer significant associations with English proficiency status.

**Conclusions and Relevance:**

In this systematic review, most of the included studies found associations between English proficiency and multiple perioperative process-of-care outcomes, but fewer associations were seen between English proficiency and clinical outcomes. Because of limitations of the existing research, including study heterogeneity and residual confounding, mediators of the observed associations remain unclear. Standardized reporting and higher-quality studies are needed to understand the impact of language barriers on perioperative health disparities and identify opportunities to reduce related perioperative health care disparities.

## Key Points

**Question:**

Is English language proficiency in adult surgical patients associated with differences in perioperative care and surgical outcomes?

**Findings:**

In this systematic review of 29 studies with 281 266 patients, surgical patients with limited English proficiency experienced reduced access, delays in care, and longer surgical admissions and were more likely to be discharged to a skilled facility than patients with English proficiency. Clinical outcomes related to mortality, postoperative complications, and unplanned readmissions showed fewer significant associations with English proficiency status.

**Meaning:**

These findings indicate that limited English proficiency is associated with several perioperative process-of-care outcomes; additional studies are needed to understand the impact of language barriers on perioperative health disparities.

## Teaser

This systematic review examines the association of limited English proficiency with perioperative care and surgical outcomes in adult patients.

## Introduction

As the proportion of surgically treated conditions continues to increase, health care disparities in the perioperative period (including the preoperative, intraoperative, and postoperative phases of a surgical admission) are of particular concern.^1,2^ Although racial and ethnic disparities in surgical access, perioperative quality of care, and surgical outcomes have been persistent and well-documented problems, much less is known about how language barriers contribute to these disparities.

Currently, 22% of people in the US speak a language other than English at home,^3^ and limited English proficiency (LEP) has been reported to be correlated with reduced access to health care, increased risk of health care–related adverse events,^4^ worse patient care experiences,^5^ reduced understanding of discharge instructions,^6^ and worse outcomes for both outpatient and hospitalized patients.^4,7,8,9,10^ There are numerous reasons to postulate that language proficiency may significantly impact perioperative health care and surgical outcomes in particular. Perioperative decisions are complex, involving consent for high-risk procedures and balancing the risk-benefit ratio of competing treatment options. The environment is fast-paced, with time pressure that is at odds with longer interpreter-assisted conversations and examinations. Furthermore, the use of untrained interpreters may compound problems with communication errors and the tendency to omit sensitive material.^11^ Finally, language barriers may limit access to, or understanding of, important preoperative or postoperative instructions.^12^

Despite extensive theoretical reasons to surmise that language barriers may impact perioperative care, a clear understanding of the current evidence is needed to inform intervention and policy efforts aimed at improving outcomes for vulnerable surgical patients with LEP. Thus, the objective of this systematic review of the literature was to examine the association of LEP with perioperative care and surgical outcomes.

## Methods

This systematic review was prospectively registered with PROSPERO (CRD42022299569)^13^ and is reported in accordance with the Preferred Reporting Items for Systematic Reviews and Meta-Analysis Protocols (PRISMA-P) guideline (eAppendix 1 in Supplement 1).^14^ The search strategy was developed in consultation with a research librarian; was performed in MEDLINE via PubMed, Embase, Web of Science, Sociological Abstracts, and CINAHL with no restriction on publication date; and consisted of Medical Subject Headings terms related to language barriers, perioperative or surgical care, and perioperative outcomes (eAppendix 2 in Supplement 1). The final search was conducted on December 7, 2022.

Original observational or experimental studies comparing perioperative care and surgical outcomes between adult patients with and without LEP were included. Outcomes across the perioperative period, including access to surgical procedures, delays in receiving surgical care, perioperative pain management, surgical admission length of stay (LOS), discharge disposition, postoperative complications, functional recovery, mortality, and postoperative readmissions, were examined (Table 1; eTable 1 in Supplement 1).^15,16,17,18,19,20,21,22,23,24,25,26,27,28,29,30,31,32,33,34,35,36,37,38,39,40,41,42,43^ The Newcastle-Ottawa Scale for cohort studies was used for risk-of-bias assessment (Table 2).^44,45^ Because of heterogeneity in analysis and reported outcomes among studies, data were not pooled for quantitative analysis. Included studies were organized by outcome of interest to facilitate data interpretation.

**Table 1.** Study Characteristics and Outcomes of Included Studies

Source	Location	Study population	Sample, No.	Data source	LEP, No. (%)	LEP definition	Age distribution by LEP, mean (SD) or median (IQR)	Sex by LEP, No. (%)		Outcome measures
								Female	Male	
John-Baptiste et al,^15^ 2004	Canada	Patients admitted to tertiary care hospital for medical and surgical conditions	44 983	Academic multicenter	6124 (13.6)	Clerk assessment of patient’s inability to communicate in English	LEP, 66.7 (NS); EP, 60.2 (NS)	LEP, 3099 (50.6); EP, 15 000 (38.6)	LEP, 3025 (49.4); EP, 23 859 (61.4)	Length of stay, in-hospital mortality
Dowsey et al,^16^ 2009	Australia	Adult patients undergoing total knee arthroplasty	278	Academic single center	41 (14.7)	Non-English spoken language and use of LEP-I	LEP, 72.0 (7.0); EP, 71.0 (9.0)	LEP, 38 (92.7); EP, 155 (65.4)	LEP, 3 (7.3); EP, 82 (34.6)	Functional outcomes
MacDonald et al,^17^ 2010	Canada	Adult patients scheduled for elective hip or knee arthroplasty	148	Community multicenter	19 (12.8)	Non-English spoken language and LEP-I required	NS	NS	NS	Length of stay
Nashed et al,^18^ 2012	US	Adult patients diagnosed with supratentorial intra-axial primary brain tumor undergoing neuro-oncologic surgery	112	Academic single center	45 (40.2)	Non-English spoken language and self-identified as Hispanic (vs White)	LEP, 51.8 (21-75); EP, 50.9 (20-87)	LEP, 22 (48.7); EP, 34 (50.7)	LEP, 23 (51.3); EP, 33 (49.3)	Delay in surgery, long-term survival
Betjemann et al,^19^ 2013	US	Adult patients with medically refractory epilepsy admitted to epilepsy monitoring unit	213	Academic single center	42 (19.7)	Non-English language preference	LEP and EP, 37 (NS)	LEP and EP, 117 (54.9)	LEP and EP, 96 (45.1)	Access to surgery
Thompson et al,^20^ 2014	US	Adult patients with unilateral mesial temporal sclerosis admitted to epilepsy monitoring unit for potential anterior temporal lobectomy	223	Academic single center	43 (19.3)	Non-English language preference	LEP and EP, 36.9 (11.6)	LEP and EP, 124 (55.6)	LEP and EP, 99 (44.4)	Delay in surgery
Tang et al,^21^ 2016	Canada	Adult patients undergoing elective isolated CABG surgery	691	Academic single center	103 (14.9)	Any patient or practitioner-reported LEP status in medical record	LEP, 67.7 (8.4); EP, 65.3 (9.1)	LEP, 28 (27.2); EP, 79 (13.4)	LEP, 75 (72.8); EP, 509 (86.6)	Length of stay
Wilbur et al,^22^ 2016	US	Patients admitted to a gynecologic oncology service for surgery	1605	Academic single center	58 (3.6)	Non-English primary language OR LEP-I required	LEP and EP, 53.2 (NS)	LEP and EP, 1605 (100)	LEP and EP, 0 (0)	Readmission
Inagaki et al,^23^ 2017	US	Patients undergoing nonemergency infrainguinal bypass surgery	261	Academic single center	51 (19.5)	Non-English primary language	LEP, 67.4 (9.8); EP, 63.1 (9.9)	LEP, 24 (47.1); EP, 78 (37.1)	LEP, 27 (52.9); EP, 132 (62.9)	Length of stay, complication, readmission
Hyun et al,^24^ 2017	Australia and New Zealand	Patients admitted with ACS event for potential intervention and/or surgery	4387	Community nationwide database	294 (6.7)	Non-English primary language	LEP, 70.9 (12.6); EP, 66.3 (14.7)	LEP, 121 (41.2); EP, 1646 (40.2)	LEP, 173 (58.8); EP, 2447 (59.8)	Length of stay, in-hospital mortality, complications, long-term mortality
Jaiswal et al,^25^ 2018	US	Patients with nonrecurrent, primary stage 0-III breast cancer receiving medical and/or surgical care	105	Community single center	25 (23.8)	Non-English primary language	LEP and EP, 4 categories of age	LEP and EP, 105 (100)	LEP and EP, 0 (0)	Delay in surgery
Feeney et al,^26^ 2019	US	Adult patients undergoing surgical oncology operations	37531	Community statewide database	3398 (9.1)	Non-English primary language, either Spanish or NENS	LEP (Spanish), 56.7 (15.8); LEP (NENS), 62.1 (14.8); EP, 59.5 (15.5)	LEP (Spanish), 1252 (71.7); LEP (NENS), 1131 (68.5); EP, 25 313 (74.2)	LEP (Spanish), 495 (28.3); LEP (NENS), 520 (31.5); EP, 8820 (25.8)	Length of stay, in-hospital mortality, readmission
Sridhar et al,^27^ 2019	US	Adult patients with stage I-IV pancreatic cancer	240	Academic single center	53 (22.1)	English is not a primary language	LEP and EP, 2 categories of age	LEP and EP, 122 (50.8)	LEP and EP, 118 (49.2)	Access to surgery, long-term survival
Feeney et al,^28^ 2019	US	Adult patients admitted for emergency surgery occurring within 2 d of admission	105 171	Community statewide database	13 716 (13.0)	Non-English primary language, either Spanish or NENS	LEP (Spanish), 42.2 (16.6); LEP (NENS), 50.5 (19.4); EP, 49.1 (19.5)	LEP (Spanish), 4961 (56.1); LEP (NENS), 2790 (57.3); EP, 52 907 (57.9)	LEP (Spanish), 3888 (43.9); LEP (NENS), 2077 (42.7); EP, 38548 (42.1)	Length of stay, in-hospital mortality, readmission
Feeney et al,^29^ 2020	US	Adult patients undergoing surgical oncologic operations	2467	Academic single center	824 (33.4)	Non-English primary language, non-English discharge instructions, or LEP-I charges	LEP, 55 (46-64); EP, 55 (45-65)	LEP, 709 (86.0); EP, 1279 (77.8)	LEP, 115 (14.0); EP, 364 (22.2)	Length of stay, complications, readmission, long-term mortality
Asokan et al,^30^ 2020	US	Adult patients with esophageal cancer undergoing consultation for treatment involving surgery, chemotherapy, or radiation therapy	266	Academic single center	43 (16.2)	Non-English primary language	LEP and EP, 2 categories of age	LEP and EP, 61 (22.9)	LEP and EP, 205 (77.1)	Access to surgery, long-term survival
Bernstein et al,^31^ 2020	US	Patients undergoing single primary hip or knee arthroplasty	12 885	Academic single center	1682 (13.1)	Non-English primary language, requiring LEP-I or LEP-N	LEP-I, 67.1 (11.5); LEP-N, 64.0 (12.1); EP, 62.9 (12.2)	LEP-I, 1125 (72.8); LEP-N, 94 (68.6); EP, 6924 (61.8)	LEP-I, 420 (27.2); LEP-N, 43 (31.4); EP, 4279 (38.2)	Length of stay, discharge disposition
Varady et al,^32^ 2020	US	Patients undergoing an orthopedic surgical procedure	17 643	Academic multicenter	1205 (6.8)	Non-English primary language	LEP and EP, 56.9 (16.9)	LEP and EP, 10 170 (57.6)	LEP and EP, 7473 (42.4)	Access to surgery
Wong et al,^33^ 2021	US	Adult patients undergoing colectomy or proctectomy	1763	Academic single center	117 (6.6)	Non-English primary language	LEP and EP, 4 categories of age	LEP and EP, 920 (52.2)	LEP and EP 843 (47.8)	Readmission
Witt et al,^34^ 2021	US	Adult patients undergoing neuro-oncologic surgery for primary brain tumor, meningioma, or brain metastasis	7402	Community statewide database	560 (7.6)	Non-English primary language, either Spanish or NENS	LEP (Spanish), 55 (44-67); LEP (NENS), 61 (52-69); EP, 60 (50-69)	LEP (Spanish), 174 (58.0); LEP (NENS), 153 (58.9); EP, 3661 (53.5)	LEP (Spanish), 126 (42.0); LEP (NENS), 107 (41.2); EP, 3181 (46.5)	Access to surgery
Schwartz et al,^35^ 2021	US	Adult trauma patients discharged to the community	1419	Community single center	237 (16.7)	Non-English spoken language(s)	LEP, 57.3 (20.5); EP 47.2 (19.1)	LEP, 82 (34.6); EP, 380 (32.1)	LEP, 155 (65.4); EP, 884 (74.8)	Pain management
Witt et al,^36^ 2021	US	Adult patients undergoing neuro-oncologic surgery for primary brain tumor, meningioma, or brain metastasis	7324	Community statewide database	554 (7.6)	Non-English primary language, either Spanish or NENS	LEP (Spanish), 55 (44-67); LEP (NENS), 61 (52-69); EP, 60 (50-69)	LEP (Spanish), 171 (57.6); LEP (NENS), 151 (58.8); EP, 3624 (53.5)	LEP (Spanish), 126 (42.4); LEP (NENS), 106 (41.2); EP, 3146 (46.5)	Length of stay, discharge disposition, in-hospital mortality, complications
Maurer et al,^37^ 2021	US	Adult patients with diverticulitis undergoing a partial colon resection	9453	Community statewide database	592 (6.3)	Non-English primary language	LEP, 58 (48-70); EP, 59 (50-69)	LEP, 305 (51.5); EP, 4707 (53.1)	LEP, 287 (48.5); EP, 4154 (46.9)	Access to surgery
Manuel et al,^38^ 2022	US	Adult patients undergoing hip or knee arthroplasty	4721	Academic single center	378 (8.0)	Non-English preferred language and request for LEP-I	LEP, 70.1 (14.2); EP, 63.5 (12.2)	LEP, 264 (69.8); EP, 2408 (55.4)	LEP, 114 (30.2); EP, 1935 (44.6)	Length of stay, discharge disposition, readmission
Aggarwal et al,^39^ 2022	Australia	Patients undergoing elective total hip arthroplasty	1412	Community multicenter	179 (12.8)	Non-English primary spoken language	LEP and EP, 67.0 (11.7)	LEP and EP, 752 (53.3)	LEP and EP, 659 (46.7)	Functional outcome
Silverstein et al,^40^ 2022	US	Patients referred for minimally invasive gynecologic surgery	1823	Academic single center	66 (3.6)	Non-English primary language	NS	LEP and EP, 1823 (100)	LEP and EP, 0 (0)	Delay in surgery
Manuel et al,^41^ 2022	US	Adult patients undergoing craniotomies for brain tumor	2232	Academic single center	150 (6.7)	Non-English primary language and use of LEP-I	LEP, 55.4 (15.4); EP, 52.8 (15.2)	LEP, 82 (54.7); EP, 1027 (49.3)	LEP, 68 (45.3); EP, 1055 (50.7)	Length of stay, discharge disposition, readmission
Stolarski et al,^42^ 2022	US	Adult patients who underwent laparoscopic sleeve gastrectomy or laparoscopic gastric bypass surgery	1662	Academic single center	671 (40.4)	Non-English preferred language OR use of LEP-I at least once during the study period	NS	LEP, 591 (88.1); EP, 882 (89.0)	LEP, 80 (11.9); EP, 109 (11.0)	Length of stay, complications, long-term outcomes, readmission
Kovoor et al,^43^ 2023	Australia	Patients admitted for general surgery	12 846	Academic multicenter	1178 (9.2)	Non-English primary language	LEP, 64.8 (19.1); EP, 53.9 (20.4)	LEP, 561 (47.6); EP, 5596 (48.0)	LEP, 617 (52.4); EP, 6072 (52.0)	Length of stay, in-hospital mortality, pain management

Abbreviations: ACS, acute coronary syndrome; CABG, coronary artery bypass surgery; EP, English proficiency; LEP, limited English proficiency; LEP-I, limited English proficiency with interpreter required; LEP-N, limited English proficiency with no interpreter required; NENS, non-English/non-Spanish; NS, nonspecified.

**Table 2.** Newcastle-Ottawa Scale Quality Assessment

Source	Selection (scale, 1-5)	Comparability (scale, 1-2)	Outcome (scale, 1-3)	Total (scale, 1-9)	Study quality^a^
John-Baptiste et al,^15^ 2004	4	1	2	7	Good
Dowsey et al,^16^ 2009	3	1	3	7	Good
MacDonald et al,^17^ 2010	4	1	3	8	Good
Nashed et al,^18^ 2012	3	0	2	5	Poor
Betjemann et al,^19^ 2013	4	2	1	7	Poor
Thompson et al,^20^ 2014	4	0	1	5	Poor
Tang et al,^21^ 2016	4	1	2	7	Good
Wilbur et al,^22^ 2016	4	2	2	8	Good
Inagaki et al,^23^ 2017	3	2	1	6	Poor
Hyun et al,^24^ 2017	4	1	2	7	Good
Jaiswal et al,^25^ 2018	3	2	2	7	Good
Feeney et al,^26^ 2019	4	2	1	7	Poor
Sridhar et al,^27^ 2019	3	0	1	4	Poor
Feeney et al,^28^ 2019	4	2	1	7	Poor
Feeney et al,^29^ 2020	4	1	2	7	Good
Asokan et al,^30^ 2020	4	0	1	5	Poor
Bernstein et al,^31^ 2020	4	1	2	7	Good
Varady et al,^32^ 2020	4	2	3	9	Good
Wong et al,^33^ 2021	4	2	2	8	Good
Witt et al,^34^ 2021	4	1	3	8	Good
Schwartz et al,^35^ 2021	4	1	2	7	Good
Witt et al,^36^ 2021	4	1	2	7	Good
Maurer et al,^37^ 2021	4	1	3	8	Good
Manuel et al,^38^ 2022	4	1	2	7	Good
Aggarwal et al,^39^ 2022	4	2	2	7	Good
Silverstein et al,^40^ 2022	4	1	2	7	Good
Manuel et al,^41^ 2022	4	2	3	9	Good
Stolarski et al,^42^ 2022	4	2	2	8	Good
Kovoor et al,^43^ 2023	4	1	2	7	Good

^a^ Based on thresholds for converting the Newcastle-Ottawa scales to Agency for Healthcare Research and Quality standards.^44^ Good quality is defined as 3 or 4 in the selection domain AND 1 or 2 in the comparability domain AND 2 or 3 in the outcome or exposure domain. Fair quality is defined as 2 in the selection domain AND 1 or 2 in the comparability domain AND 2 or 3 in the outcome or exposure domain. Poor quality is defined as 0 or 1 in the selection domain OR 0 in the comparability domain OR 0 or 1 in the outcome exposure domain.

## Results

### Search Results

Our database search yielded 2230 unique records (Figure). After title and abstract review, 70 manuscripts were identified for full-text review. After full-text review, 29 studies^15,16,17,18,19,20,21,22,23,24,25,26,27,28,29,30,31,32,33,34,35,36,37,38,39,40,41,42,43^ met the inclusion criteria (eTable 2 in Supplement 1).

**Figure.**
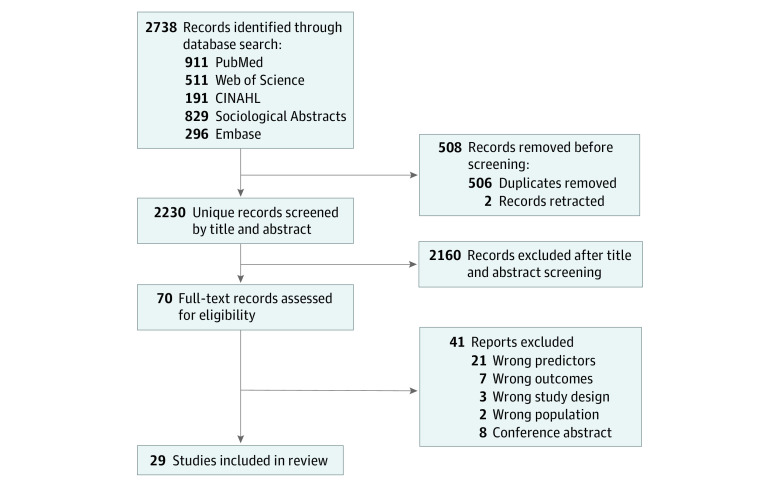
Flow Diagram

### Study Characteristics

All included studies took place in English-speaking countries, including the US, Australia, New Zealand, and Canada (Table 1). Although our search had no date restriction, studies in our sample were published between 2004 and 2023, with most published from 2019 to 2022 (eFigure 1 in Supplement 1).

All included studies were observational, and 26 of 29 studies^15,18,19,20,21,22,23,25,26,27,28,29,30,31,32,33,34,35,36,37,38,39,40,41,42,43^ were retrospective. All were cohort studies except for 1 cross-sectional study. Studies reported findings from academic hospital settings and community hospitals. Eighteen of 29 studies^16,18,19,20,21,22,23,25,27,29,30,31,33,35,38,40,41,42^ were performed at a single center, whereas 5 studies^15,17,32,39,43^ were multicenter and 6 studies^24,26,28,34,36,37^ used a statewide or nationwide database (Table 1).

Eighteen studies^16,17,21,22,23,26,28,29,31,32,33,34,36,38,39,41,42,43^ evaluated a patient cohort that underwent surgical admission, whereas 11 studies^15,18,19,20,24,25,27,30,35,37,40^ contained patient cohorts that underwent evaluation for surgical intervention from an outpatient, general hospital admission, or emergency department (ED) setting. Median study cohort size was 1763 (IQR, 266-7402), with a median LEP cohort size of 179 (IQR, 51-671).

The 29 included studies had a total of 281 266 patients (mean [SD] age, 57.2 [10.0] years; 121 772 [43.3%] male and 159 240 [55.6%] female). Cohort summary statistics are presented in Table 1. Most studies reported race and/or ethnicity, and 11 studies^19,22,23,26,28,29,32,33,38,41,42^ controlled for race and/or ethnicity in multivariable analysis. Surgical specialties included general, oncologic, orthopedic, neurologic, thoracic, gynecologic, urologic, head and neck, trauma, and vascular surgery.

### Defining LEP

This review found substantial variation in the methods used to define language proficiency and LEP. Most included studies^17,18,19,20,23,24,25,26,27,28,30,32,33,34,35,36,37,39,40,43^ defined LEP using self-report measures that were documented in electronic medical records. Many studies^15,16,17,18,19,20,21,22,23,24,25,27,28,29,30,31,32^ used a non-English preferred language as a proxy for LEP. Nine studies confirmed LEP using multiple methods, such as both self-report of non-English preferred language and documentation of interpreter requirements,^16,18,21,22,29,38,41,42^ both self-report of non-English preferred language and self-identification of Hispanic ethnicity,^18^ cross-checking self-reported preferred language by both electronic records and discharge instructions combined with accrual of interpreter service charges,^29^ or some other method.^21,31^ Some studies evaluated LEP via structural interview by research clerks^15^ or a patient-initiated screening tool.^17^ Five studies^26,28,34,36,37^ subcategorized the preferred language of patients with LEP into Spanish vs others. Studies that solely used ethnicity or country of birth as a proxy for language proficiency were excluded from this systematic review.

### Risk-of-Bias Assessment

The risk-of-bias assessment using the Newcastle-Ottawa Scale and Agency for Healthcare Research and Quality thresholds for study quality found 21 studies^15,16,17,21,22,24,25,29,31,32,33,34,35,36,37,38,39,40,41,42,43^ to be of good quality and 8 studies^18,19,20,23,26,27,28,30^ to be poor quality (Table 2).^44^ Poor scores were most commonly due to concerns with comparability (eg, analyses did not adequately control for confounders, such as race and ethnicity).

### Access to Surgical Care

Six included studies^19,27,30,32,34,37^ assessed access to surgical care (eTable 1 in Supplement 1). Of these, 3 studies^19,27,30^ directly assessed receipt of surgery. One study^34^ investigated emergency vs elective admission, and another study^37^ assessed admission to a low-volume vs high-volume hospital, which indirectly represented reduced access. One study^32^ used a proxy variable (utilization of the electronic patient portal) to demonstrate differential access to surgical care. Four^19,32,34,37^ of these 6 studies found LEP to be significantly associated with reduced access to care in adjusted analysis. However, 1 of these studies^34^ revealed a significant association only in patients with non–Spanish-speaking LEP. Two studies^27,30^ performed only unadjusted analyses and identified no association between LEP and limited access to care.

### Delays in Surgical Care

Four studies^18,20,25,40^ evaluated the association between LEP and delays in receiving surgical care (eTable 1 in Supplement 1). Studies defined surgical delay uniquely: time from the date of diagnosis to date of surgery,^18^ time from hospital admission to the date of surgery,^18,20^ time from the date of diagnosis to the date of treatment,^25^ and time from the date of referral to the date of surgical department initial encounter.^25,40^ Two studies^20,25^ reported significant delays in care among patients with LEP, whereas 1 study^18^ reported no difference. The remaining study was equivocal, reporting significant delays in care for patients with LEP in the more recent subcohort analysis, but such association was not significant in the historical subcohort.^40^

### Surgical Admission LOS

Fourteen studies^15,17,21,23,24,26,28,29,31,36,38,41,42,43^ evaluated the association between LEP and LOS (eTable 1 in Supplement 1). There was a high degree of heterogeneity in processing and analyzing the LOS variable. Two studies^17,43^ dichotomized LOS into either long or short LOS using a cutoff at 5 or 7 days each. Although most studies assessed total LOS defined as time from surgical hospital admission to discharge, 2 of 14 studies^23,36^ assessed postoperative LOS defined as time from end of surgical procedure to discharge.

Five studies found significantly prolonged total LOS in patients with LEP admitted for surgical procedures, including for coronary artery bypass graft,^15^ craniotomy,^15,41^ intestinal and rectal surgery,^15^ hip arthroplasty,^15,17,31,38^ and knee arthroplasty. Of these 5 studies, 1 study^15^ demonstrated mixed results depending on surgical cohorts. One study^36^ of neuro-oncology patients showed significantly longer postoperative LOS in patients with non–Spanish-speaking LEP but no association with total LOS. Eight studies^21,23,24,26,28,29,42,43^ found no association between LEP and total or postoperative LOS. A single study^28^ found that patients with Spanish-speaking LEP had shorter total LOS for appendectomy and patients with either Spanish-speaking or non–Spanish-speaking LEP had shorter total LOS for adhesiolysis compared with English-proficient patients. However, when looking at a subcohort of high-risk procedures in this same study, Spanish-speaking and non–Spanish-speaking patients with LEP had increased LOS compared with English-proficient patients.

### Discharge Disposition

Four studies^31,36,38,41^ evaluated the association between LEP and postoperative discharge disposition (eTable 1 in Supplement 1). Patients with LEP were more likely to be discharged to skilled facilities vs home after total joint arthroplasty^38^ and craniotomy.^41^ In another study,^31^ patients with LEP who needed an interpreter had a significantly higher chance of discharge to skilled facilities after total joint arthroplasty than those with English proficiency, whereas patients with LEP who did not require an interpreter had no such association. In a study^36^ of neuro-oncologic surgery patients, Spanish-speaking LEP was associated with lower odds of discharge to skilled facilities, although no association was observed between non–Spanish-speaking LEP and discharge disposition.

### Mortality

Six studies^15,24,26,28,36,43^ evaluated the association between LEP and perioperative in-hospital mortality (eTable 1 in Supplement 1). Five of these studies found no significant association between LEP and in-hospital mortality; only 1 study^15^ found a subcohort of patients with LEP undergoing aortic aneurysm repair who had significantly higher in-hospital mortality than those with English proficiency in adjusted analysis.

Five studies^18,24,27,29,30^ evaluated the association between LEP and postoperative all-cause mortality (or overall survival). Two studies^24,29^ assessed the binary outcome of all-cause mortality, and 3 studies^18,27,30^ looked at overall survival time to capture mortality data. Four studies^18,24,29,30^ identified no association between LEP and all-cause mortality; however, 1 study^27^ detected significantly longer median overall survival in a subcohort of patients with stage III to IV pancreatic cancer with LEP in unadjusted analysis. Overall, few studies^15,27^ found a significant association between LEP and mortality in adjusted analyses.

### Complications

Five studies assessed postoperative complications, including wound infection,^23^ adverse graft event,^23^ major adverse cardiac events,^24^ major morbidities based on the National Surgical Quality Improvement Program risk calculator criteria,^29^ and general short-term postoperative complications.^36,42^ Only 1 included study^36^ observed a significant association between LEP and complications. This study found higher odds of developing complications after neuro-oncologic surgery in patients with non–Spanish-speaking LEP compared with English-proficient patients.

### Readmissions

Nine studies^22,23,26,28,29,33,38,41,42^ evaluated the association between LEP and unplanned hospital readmissions after surgical hospitalization. Seven studies^22,23,26,28,38,41,42^ assessed hospital readmissions within 7 days, 30 days, or 1 year after discharge (eTable 1 in Supplement 1). Four studies^23,27,40,42^ investigated ED visits within 30 days or 1 year after discharge.

Most studies found no association between LEP and readmissions. A single study^22^ identified significantly more 30-day readmissions in patients with LEP than English proficiency who underwent gynecologic oncology surgery. Of the 4 studies that addressed ED visits, 2 studies^23,29^ found no association between LEP and ED visits after infrainguinal bypass or oncologic surgery, 1 study^33^ reported a significantly higher rate of ED visits among patients with LEP after proctocolectomy, and 1 study^42^ demonstrated fewer ED visits among patients with LEP after gastric surgery.

### Perioperative Pain Management

A single study^35^ in our review evaluated the association between LEP and receipt of a discharge opioid prescription after surgical care, finding that patients with LEP were less likely to receive a discharge opioid prescription, and even if they did, the oral morphine equivalent amount was lower for patients with LEP than English proficiency. One other study^43^ found general surgical patients with LEP had significantly lower median pain scores recorded during their admission than patients with English proficiency.

### Functional Outcomes

Two studies^16,39^ evaluated the association between LEP and postoperative function after arthroplasty procedures. One study^16^ found significantly lower 12-month postoperative functional status using International Knee Society scores among patients with LEP vs English-proficient patients. Another study^39^ found that patients with LEP had significantly lower Oxford hip scores after total hip arthroplasty, indicating worse function and pain. However, the association between LEP and patient-rated improvement scores failed to reach statistical significance in the same study. A single study^42^ reported no difference in mean excess weight loss 1 year after laparoscopic sleeve gastrectomy or gastric bypass between individuals with and without LEP.

## Discussion

This systematic review included 29 studies^15,16,17,18,19,20,21,22,23,24,25,26,27,28,29,30,31,32,33,34,35,36,37,38,39,40,41,42,43^ examining the association between LEP and a broad range of perioperative care and surgical outcomes (eFigure 2 in Supplement 1). Outcomes corresponding to efficiency in the perioperative process of care, such as accessibility, timely delivery of care, LOS, and discharge disposition, demonstrated the most consistent association with LEP status. On the other hand, clinical outcomes, such as postoperative complications, unplanned readmissions and ED visits, and mortality, were less frequently associated with LEP. Only a small number of studies examined differences in perioperative pain management or postoperative functional outcomes.

The substantial variation in methods used to define LEP in included studies was notable. Inconsistent ascertainment of our primary exposure of interest led to heterogeneity that limited our ability to accurately estimate the true association of language barriers or draw strong conclusions from the findings. Concerns about potential misclassification of patients by English proficiency status are also valid and can result in underestimation or overestimation of the association. Validated methods for determining the LEP status of patients should be used by future investigators, including asking patients their preferred language combined with the question, “How well do you speak English?”^46^ or combining non–English-preferred language with a request for interpreter services.^38^ In addition, data on health care team language proficiency and family member or professional interpreter use were not collected by most included studies but would be useful.

The heterogeneity of study settings, surgical subspecialties, and outcome measurements also prevented pooling of data for meta-analysis. We are, nonetheless, able to provide important data about the quality and quantity of existing literature and to report the directionality of evidence.

Most studies^18,19,20,22,23,25,27,29,30,31,33,35,38,40,41,42^ included in this review reported the experience of a single hospital or health care system from 1 of 7 states in the US, limiting generalizability. However, a few studies^26,28,34,36,37^ examined a large statewide (New Jersey) database of community hospital discharges. Although the extent to which this source of hospitalization data provides consistent and standardized measures of LEP status is still unknown, investigators were able to account for hospital- or physician-level effects, enabling more complex analyses to unveil intrahospital or intraphysician correlations.^26,28^

Furthermore, with only observational evidence available and likely residual confounding, we cannot infer causation; we can only note the frequently observed associations between LEP and perioperative process outcomes. We recognize that LEP status coexists with, and is extremely difficult to disentangle from, many other social determinants of health, including racism, immigration status, social segregation, occupational hierarchy, economic security, insurance coverage, health literacy, and cultural differences. Although most included studies conducted adjusted analyses to reduce confounding between LEP and observed outcomes, sets of adjustment variables were highly variable and driven by setting, surgical procedure, or population. Although some studies adjusted for race and ethnicity, which are known to be correlated with health outcomes in English-speaking countries, many studies did not include these covariates or did not adjust for them for various statistical reasons: multicollinearity,^24^ model fitting,^34^ stepwise selection,^35^ decision a priori,^36^ unadjusted analysis only,^20,25,27,30^ or other reasons.^37^ Simultaneously, however, although risk of unmeasured confounding and inconsistent adjustment in the included observational studies limit our ability to draw conclusions about exposure-outcome relationships, an experimental study design (such as randomized clinical trials) may be considered unethical because there is significant evidence that lack of interpretation services in health care settings is linked to worse outcomes and safety issues.^11,47^ The current lack of inclusion of preferred language and interpreter use data in national databases diminish investigators’ abilities to include language barriers in large multivariable models examining predictors of perioperative outcomes, generate more generalizable findings, more completely control for confounding factors, and detect smaller effect sizes. Unsurprisingly, in unadjusted analyses (eTable 3 in Supplement 1), which did not attempt to isolate the impact of language barriers from common confounders, such as insurance status and comorbidities, patients with LEP were more likely to experience more unfavorable perioperative and surgical outcomes than English-proficient patients.

Despite our inability to comment on mediators of the observed associations, this systematic review corroborates findings from other reviews examining the association of English proficiency with various health care outcomes. Woods et al^48^ reviewed 26 studies and reported that LEP was associated with inpatient LOS in 8 of 17 studies and not associated with LOS in 9 of 17 studies, with directionality toward prolonged LOS for patients with LEP. No association was found between LEP and mortality or clinical complications in hospitalized patients of both medical and surgical services in this review. Hsueh et al^49^ and Diamond et al^50^ reviewed the association of physician-patient language concordance with health outcomes in primary care and hospital settings and found that language discordance was associated with lower-quality medical communication, reduced understanding, reduced health care access and utilization, suboptimal patient-physician interactions, and worse clinical outcomes, including glycemic control and adverse medications events.

Most recently, Luan-Erfe et al^51^ attempted to review the association of LEP with perioperative and perianesthetic outcomes. They included 10 studies, most of which examined pediatric populations or nonsurgical cohorts and none of which met the inclusion criteria of this systematic review (eTable 2 in Supplement 1). The results of the review by Luan-Erfe et al^51^ were mixed, with limited ability to draw conclusions because of the small number of studies per outcome examined. Overall, the authors determined that language barriers were negatively associated with patient-centered perioperative outcomes, such as less patient participation in the informed consent process, insufficient understanding of discharge instructions, delayed or infrequent pain assessments, and higher likelihood of ED visits after discharge; however, LEP was not associated with access to appointments, adequacy of procedure preparation, pain management, or postdischarge follow-up encounters.^51^ Our findings complement the findings of Luan-Erfe et al^51^ and more fully characterize the association of language barriers with the care and outcomes of surgical patients with LEP. These reviews all agree that LEP may be independently correlated with efficiency outcomes and patient-centered aspects of care regardless of health care settings; however, evidence of a strong influence of LEP on clinical outcomes is not observed.

There is a clear need for additional research on the impact of LEP and perioperative and surgical care. An important finding of the current systematic review is that many individual centers observe disparities in outcomes by LEP status, which warrants inclusion of language variables in larger surgical outcome studies. However, to perform higher-quality studies, greater consistency and rigor are needed in the collection of language and interpreter use data and in the ascertainment of perioperative measures of interest. Large nationwide databases should include routine collection of preferred language and English proficiency to facilitate this research as well as investigate concerns of specific language minority groups and assess less common outcomes. Finally, many clinically important underexplored outcomes, such as long-term functional recovery and perioperative pain management, should be examined. Importantly, additional investigations are needed to establish an understanding of the pathways by which language barriers may impact perioperative health outcomes so that interventions to reduce observed disparities can be created.

### Strengths and Limitations

 A strength of this systematic review is its use of rigorous methods. However, the results should be interpreted in the context of its limitations. Only peer-reviewed published original research is included, so the findings reported could be subject to publication bias. Because of the heterogeneity of included studies with regard to study setting, surgical subspecialty, outcome measurement, statistical methods, and measure of association, we were unable to pool data to perform a meta-analysis (eMethods in Supplement 1). We are, nonetheless, able to report directionality of evidence. There was variation in definition and ascertainment of LEP status, and a standardized method of collecting language and interpreter data is needed. The way that outcomes of interest were measured in the included studies may allow for variations in residual confounding effects, which impede our ability to assess differences for patients with LEP. Where numerous outcomes and comparisons were evaluated, there is a risk that some findings might be statistically significant by chance. Additionally, the quality of evidence ratings provided by the Newcastle-Ottawa Scale are largely subjective, and some might disagree with our assessments.

## Conclusions

In this systematic review, many of the included studies found associations between LEP and multiple perioperative process-of-care outcomes, but fewer associations were seen between English proficiency and clinical outcomes. Limited data found lower perioperative opioid use and worse functional recovery among patients with LEP. Because of study limitations, including heterogeneity and residual confounding, mediators of the observed associations remain unclear. Standardized reporting and larger, higher-quality studies are needed to understand the effect of language barriers on perioperative health disparities and provide the basis for interventions to reduce perioperative health care disparities in the increasing LEP population.

## Notes

Supplement 1. **eAppendix 1.** Methodological Details **eAppendix 2.** Detailed Search Strategy
eReferences
**eTable 1.** Study Outcomes, Methods, and Results by Category **eTable 2.** Decision for Inclusion and Exclusion **eTable 3.** Unadjusted Results **eFigure 1.** Articles per Year that Met Inclusion Criteria **eFigure 2.** Perioperative Outcomes Represented

Supplement 2.
Data Sharing Statement
