# Supplementary material for: Association of Language Barriers With Perioperative and Surgical Outcomes: A Systematic Review
Source: JAMA Netw Open. 2023 Jul 11;6(7):e2322743. doi: 10.1001/jamanetworkopen.2023.22743 (PMC10336626; doi:10.1001/jamanetworkopen.2023.22743)
Supplement: Supplement 1. — eAppendix 1. Methodological Details eAppendix 2. Detailed Search Strategy eReferences eTable 1. Study Outcomes, Methods, and Results by Category eTable 2. Decision for Inclusion and Exclusion eTable 3. Unadjusted Results eFigure 1. Articles per Year that Met Inclusion Criteria eFigure 2. Perioperative Outcomes Represented [file jamanetwopen-e2322743-s001.pdf]

Supplementary Online Content

Joo H, Fernández A, Wick EC, Moreno Lepe GM, Manuel SP. Association of language barriers with perioperative and surgical outcomes: a systematic review. *JAMA Netw Open*. 2023;6(7):e2322743. doi:10.1001/jamanetworkopen.2023.22743

eAppendix 1. Methodological Details
eAppendix 2. Detailed Search Strategy
eReferences
eTable 1. Study Outcomes, Methods, and Results by Category
eTable 2. Decision for Inclusion and Exclusion
eTable 3. Unadjusted Results
eFigure 1. Articles per Year That Met Inclusion Criteria
eFigure 2. Perioperative Outcomes Represented

This supplementary material has been provided by the authors to give readers additional information about their work.

eAppendix 1. Methodological Details

This systematic review is reported in accordance with the Preferred Reporting Items for Systematic Reviews and Meta-Analysis Protocols (PRISMA-P) guidelines.[1] We sought to answer the question: "Is limited English proficiency in adult patients associated with differences in perioperative care and surgical outcomes, as compared to English proficient patients?" We specified the research question and prospectively registered our research protocol with PROSPERO (CRD42022299569) prior to the initiation of the search. [2]

Search Strategy
A search strategy was developed in conjunction with a research librarian (Josephine Tan, MLIS) and consisted of Medical Subject Headings (MeSH) terms related to language barriers, perioperative or surgical care, and perioperative outcomes as listed in Appendix 1. We performed searches in MEDLINE via PubMed, EMBASE, Web of Science, Sociological Abstracts, and CINAHL with no restriction on publication date. The final search was conducted on December 7, 2022. Citations listed in bibliographies of articles that met eligibility criteria, including any relevant systematic reviews and meta-analyses, were also reviewed. We also consulted experts in the field of language barrier research for additional article suggestions. Only studies in English and conducted in English speaking countries were included.

Study Selection Criteria
Inclusion criteria included original observational or experimental studies (cohort, case-control, cross-sectional, and randomized clinical trials) comparing perioperative care and surgical outcomes between adult (18+ years) patients with and without limited English proficiency. Studies must have an identified control group that is English proficient and findings based on primary or secondary data analysis. Studies were conducted in English speaking countries, and published in an English-language, peer-reviewed journal.

Exclusion criteria included: studies with only qualitative data, case reports, perspective pieces, editorials, information in books, letters, dissertations, lectures, conference abstracts, incomplete articles; studies that define communication barriers as other than linguistic barriers (e.g., cognitive deficit, speech-language pathology, physical handicap); studies that do not consider outcomes following a surgical or anesthetic intervention (e.g., primary care services, preventative care); studies without a comparison group of English proficient patients; studies did not specify limited English proficiency status as a primary/secondary predictor or a part of prediction sets *a priori*; studies not conducted in English speaking countries; studies in pediatric populations (i.e. included participants <18 years of age); non-human research; and studies with outcomes in obstetrics or labor and delivery.

Study outcomes
We examined outcomes across the perioperative period (including the preoperative, intraoperative, and postoperative phases of a surgical admission). Reported outcomes included access to surgical procedures, delays in receiving surgical care, perioperative pain management, length of stay, discharge disposition, postoperative complications, functional recovery, mortality, and hospital readmissions (Table 1, eTable 1). There were no included studies that reported outcomes related to anesthetic or surgical technique. We sought outcome data pertaining to each perioperative domain regardless of time points and analytic methods used.

Data Extraction
Search results were organized and duplicates removed using the reference manager, Zotero (https://www.zotero.org). Reference files were then imported into a systematic review organization software, Rayyan (http://rayyan.qcri.org). Initial title and abstract screening was completed by two researchers (S.M and G.M.) independently. Eligibility disagreements were resolved by consensus. Manuscripts deemed potentially eligible for inclusion then underwent full text screening by two independent reviewers (S.M. and H.J.). Any disagreements over inclusion were resolved by consensus and in consultation with a third researcher (A.F.). All reasons for exclusion during the full text screen were recorded based on the inclusion and exclusion document (Supplementary Table 1).

Two independent investigators (S.M. and H.J.) extracted data from the selected studies. The investigators re-reviewed each study when required to meet consensus on discordance. The following data were extracted from all included studies using a standardized extraction form: publication information/year, study location, study design/methodology, study population, setting, type of anesthetic/surgical intervention, how limited English

© 2023 Joo H et al. *JAMA Network Open.*

proficiency was defined, whether studies further sub-categorized limited English proficiency, whether studies included race and/or ethnicity, comparator group, covariates used to control for socioeconomic status, other covariates, outcomes of interest, the magnitude and direction of associations between LEP and the outcome measures including but not limited to odds ratio, hazard ratio, means ratio, incidence rate ratio, relative risk, and absolute mean difference with 95% confidence intervals. If additional confirmation of data was necessary, we searched for supplementary documents and reports from the same study. Data extraction results were processed and documented in Microsoft Excel (https://office.microsoft.com/excel) tables. The studies were grouped by outcome measures studied.

Risk of Bias Assessment

The Newcastle-Ottawa Scale (NOS) for cohort studies was selected for bias assessment because all included studies in this review utilized a non-randomized observational design.[3] The NOS is a validated tool with established inter-rater reliability that uses a rating system for study group selection, cohort comparability, and assessment of the exposure or outcome of interest. The maximum rating is 9 stars, with higher-quality studies receiving more stars. Two authors (S.M. and H.J.) independently assessed the risk of bias of each included study with differences resolved by consensus.

Data Synthesis

Due to lack of homogeneity in analysis and outcomes among studies, we were unable to pool data to synthesize in a quantitative analysis. Instead, studies were grouped, analyzed, and presented using a narrative approach. If applicable, measures of association were converted so that they represent the relationship between LEP and outcome domains with EP patients being the reference group. Assessment of certainty of evidence using the GRADE system was not conducted due to inadequate quality and inability to quantitatively synthesize data by outcome category.[4] Alternatively, we organized included studies by outcome of interest to facilitate data interpretation (Figure 2).

Limitations

This systematic review design has several limitations. Only peer-reviewed published original research is included, so the findings reported could be subject to publication bias as any relevant unpublished studies would not be included. Due to the heterogeneity of included studies with regards to study setting, surgical subspecialty, outcome measurement, statistical methodology, and measure of association, we were unable to pool data to perform a meta-analysis or otherwise directly compare findings to each other. For example, though multiple studies evaluated length of stay (LOS), the definition of LOS varied across studies (sometimes defined from presurgical admission to discharge, and other times only included post-procedure hospitalization time). the variable was processed differently (one study dichotomized LOS by an arbitrary cutoff, others converted LOS to quartiles or logarithm, and others treated LOS as a continuous variable), and measures of association used to describe effect size estimates were widely variable (including means ratio, odds ratio, incidence rate ratio, and absolute difference in mean). We are, none-the-less, able to report directionality of evidence. A wide variation in ascertainment of LEP exposure may have influenced associations that were found in this systematic review. While some studies used only non-English primary or preferred language to define LEP, other studies used additional measures to verify limited ability to communicate in English in the healthcare setting. In line with this variability, a vast majority of included studies did not provide information regarding how hospitals or health systems managed language barriers for LEP patients. It is probable that the observational studies included in this review were unable to control for all potential confounding variables, such as unmeasured differences between the EP and LEP cohorts (i.e., patient-provider language concordance). The way that outcomes of interest were measured in the studies allowed for variations in residual confounding effects, which impeded our ability to assess differences for patients with LEP. Both inapplicability of quantitative synthesis and possibility of residual confounding hinder assessment of certainty of evidence and complicate interpretation of the body of evidence. Where numerous outcomes and comparisons were evaluated, there is a risk that some findings might be statistically significant by chance. This is a particular concern with studies that constructed multivariable prediction models[5–7] or conducted preliminary multiple comparisons prior to establishing final risk score models.[8] Moreover, the quality of evidence ratings provided by NOS are largely subjective and some might disagree with our assessments. Lastly, qualitative studies describing subjective experience of LEP patients were not included *a priori* although some studies suggest there may be a link between language barrier and shared decision making.

© 2023 Joo H et al. *JAMA Network Open.*

eAppendix 2. Detailed Search Strategy

Key search term categories:
language barriers (eg, "language barriers," "language proficiency," "communication barriers," "English proficiency," "non-English," "limited English proficiency," "linguistic disparity");
Perioperative or surgical care (eg, "preoperative," "intraoperative," "postoperative," "postoperative care," "perioperative," "anesthesia," "anesthesiologist," "surgeon," "Surgical Procedure*," "operation," "operating room," "postanesthesia care unit," "PACU")
Perioperative outcomes (eg, "Access," "Disparity," "Intraoperative Complications," "Postoperative Complications," "complications," "morbidity," "morbidities," "Pain," "Nausea," "Symptom management," "Reoperation," "Survival Rate," "Mortality," "Length of Stay," "Readmission," "Treatment Outcome," "Outcome Assessment," "Outcomes Research," "Outcome Study," "Outcome Measures")

Final search completed December 7, 2022

PubMed
("language barriers" OR "language proficiency" OR "communication barriers" OR "communication barriers"[mesh] OR "English proficiency" OR "limited English proficiency" OR "non-English" OR "linguistic disparity") AND (preoperative OR intraoperative OR postoperative OR "postoperative care"[mesh] OR perioperative OR anesthesia OR anesthesiologist OR surgeon OR "Surgical Procedure" OR surgery OR operation OR "operating room" OR "postanesthesia care unit" OR PACU) AND (morbidity OR morbidity[mesh] OR "length of stay" OR "length of stay"[mesh] OR disparity OR "Intraoperative Complications" OR "Postoperative Complications" OR complications OR Pain OR Nausea OR "Symptom management" OR Reoperation OR "Survival Rate" OR Mortality OR Readmission OR "Treatment Outcome" OR "Outcome Assessment" OR "Outcomes Research" OR "Outcome Study" OR "Outcome Measures")

Filters applied: Humans, English

Web of Science
("language barriers" OR "language proficiency" OR "communication barriers" OR "English proficiency" OR "limited English proficiency" OR "non-English" OR "linguistic disparity") AND (preoperative OR intraoperative OR postoperative OR "postoperative care" OR perioperative OR anesthesia OR anesthesiologist OR surgeon OR "Surgical Procedure" OR surgery OR operation OR "operating room" OR "postanesthesia care unit" OR PACU) AND (morbidity OR "length of stay" OR disparity OR "Intraoperative Complications" OR "Postoperative Complications" OR complications OR Pain OR Nausea OR "Symptom management" OR Reoperation OR "Survival Rate" OR Mortality OR Readmission OR "Treatment Outcome" OR "Outcome Assessment" OR "Outcomes Research" OR "Outcome Study" OR "Outcome Measures")

Filters applied

CINAHL
("language barriers" OR "language proficiency" OR "communication barriers" OR "English proficiency" OR "limited English proficiency" OR "non-English" OR "linguistic disparity") AND (preoperative OR intraoperative OR postoperative OR "postoperative care" OR perioperative OR anesthesia OR anesthesiologist OR surgeon OR "Surgical Procedure" OR surgery OR operation OR "operating room" OR "postanesthesia care unit" OR PACU) AND (morbidity OR "length of stay" OR disparity OR "Intraoperative Complications" OR "Postoperative Complications" OR complications OR Pain OR Nausea OR "Symptom management" OR Reoperation OR "Survival Rate" OR Mortality OR Readmission OR "Treatment Outcome" OR "Outcome Assessment" OR "Outcomes Research" OR "Outcome Study" OR "Outcome Measures")

Filters applied: English language

Sociological Abstracts
("language barriers" OR "language proficiency" OR "communication barriers" OR "English proficiency" OR "limited English proficiency" OR "non-English" OR "linguistic disparity") AND (preoperative OR intraoperative OR postoperative OR "postoperative care" OR perioperative OR anesthesia OR anesthesiologist OR surgeon OR

© 2023 Joo H et al. *JAMA Network Open.*

“Surgical Procedure” OR surgery OR operation OR “operating room” OR “postanesthesia care unit” OR PACU) AND (morbidity OR “length of stay” OR disparity OR “Intraoperative Complications” OR “Postoperative Complications” OR complications OR Pain OR Nausea OR “Symptom management” OR Reoperation OR “Survival Rate” OR Mortality OR Readmission OR “Treatment Outcome” OR “Outcome Assessment” OR “Outcomes Research” OR “Outcome Study” OR “Outcome Measures”)

Filters applied: Peer-reviewed, English language

EMBASE

('language barriers' OR 'language proficiency'/exp/mj OR 'communication barriers'/exp/mj OR 'english proficiency'/exp/mj OR 'limited english proficiency'/exp/mj OR 'non-english' OR 'linguistic disparity') AND ('preoperative' OR 'intraoperative' OR 'postoperative' OR 'postoperative care'/exp/mj OR 'perioperative' OR 'anesthesia'/exp/mj OR 'anesthesiologist'/exp/mj OR 'surgeon'/exp/mj OR 'surgical procedure'/exp/mj OR 'surgery'/exp/mj OR 'operation'/exp/mj OR 'operating room'/exp/mj OR 'postanesthesia care unit'/exp/mj OR 'pacu') AND ('morbidity'/exp/mj OR 'length of stay'/exp/mj OR 'disparity'/exp/mj OR 'intraoperative complications'/exp/mj OR 'postoperative complications'/exp/mj OR 'complications'/exp/mj OR 'pain'/exp/mj OR 'nausea'/exp/mj OR 'symptom management'/exp/mj OR 'reoperation'/exp/mj OR 'survival rate'/exp/mj OR 'mortality'/exp/mj OR 'readmission'/exp/mj OR 'treatment outcome'/exp/mj OR 'outcome assessment'/exp/mj OR 'outcomes research'/exp/mj OR 'outcome study' OR 'outcome measures') AND [English]/lim

Filters applied: Humans, English language

© 2023 Joo H et al. *JAMA Network Open.*

eReferences

1. Page MJ, Moher D, Bossuyt PM, et al. PRISMA 2020 explanation and elaboration: updated guidance and exemplars for reporting systematic reviews. *BMJ*. 2021;372:n160. doi:10.1136/bmj.n160

2. Manuel SP, Fernández A, Bucklin A, Moreno Lepe G, Wick E. The Impact of Language Barriers on Perioperative Care and Surgical Outcomes: A Systematic Review Proposal. Accessed February 1, 2023. https://www.crd.york.ac.uk/prospero/display_record.php?ID=CRD42022299569

3. Wells GA, Shea B, O'Connell D, Peterson J, Welch V, Losos M, Tugwell P. The Newcastle-Ottawa Scale (NOS) for Assessing the Quality of Nonrandomised Studies in Meta-Analyses. Accessed February 1, 2023. https://www.ohri.ca/programs/clinical_epidemiology/oxford.asp

4. Holger J Schünemann, Julian PT Higgins, Gunn E Vist, Paul Glasziou, Elie A Akl, Nicole Skoetz, Gordon H Guyatt; on behalf of the Cochrane GRADEing Methods Group (formerly Applicability and Recommendations Methods Group) and the Cochrane Statistical Methods Group. Completing 'Summary of findings' tables and grading the certainty of the evidence. In: *Cochrane Handbook for Systematic Reviews of Interventions Version 6.3*. Accessed February 3, 2023. https://training.cochrane.org/handbook/current/chapter-14

5. MacDonald V, Ottem P, Wasdell M, Spiwak R. Predictors of prolonged hospital stays following hip and knee arthroplasty. *International Journal of Orthopaedic & Trauma Nursing*. 2010;14(4):198-205. doi:10.1016/j.ijotn.2010.06.001

6. Wilbur MB, Mannschreck DB, Angarita AM, et al. Unplanned 30-day hospital readmission as a quality measure in gynecologic oncology. *Gynecol Oncol*. 2016;143(3):604-610. doi:10.1016/j.ygyno.2016.09.020

7. Aggarwal A, Naylor JM, Adie S, Liu VK, Harris IA. Preoperative Factors and Patient-Reported Outcomes After Total Hip Arthroplasty: Multivariable Prediction Modeling. *J Arthroplasty*. 2022;37(4):714-720.e4. doi:10.1016/j.arth.2021.12.036

8. Silverstein RG, McClurg AB, Moore KJ, Fliss MD, Louie M. Patient Characteristics Associated with Access to Minimally Invasive Gynecologic Surgery: Changes during the COVID-19 Pandemic. *J Minim Invasive Gynecol*. 2022;29(9):1110-1118. doi:10.1016/j.jmig.2022.06.016

9. Sridhar P, Misir P, Kwak H, et al. Impact of Race, Insurance Status, and Primary Language on Presentation, Treatment, and Outcomes of Patients with Pancreatic Adenocarcinoma at a Safety-Net Hospital. *Journal of the American College of Surgeons*. 2019;229(4):389-396. doi:10.1016/j.jamcollsurg.2019.05.027

10. Asokan S, Sridhar P, Qureshi MM, et al. Presentation, Treatment, and Outcomes of Vulnerable Populations With Esophageal Cancer Treated at a Safety-Net Hospital. *Seminars in Thoracic & Cardiovascular Surgery*. 2020;32(2):347-354. doi:10.1053/j.semtcvs.2019.12.008

11. Betjemann JP, Thompson AC, Santos-Sánchez C, Garcia PA, Ivey SL. Distinguishing language and race disparities in epilepsy surgery. *Epilepsy & Behavior*. 2013;28(3):444-449. doi:10.1016/j.yebeh.2013.06.020

12. Varady NH, d'Amonville S, Chen AF, d'Amonville S. Electronic Patient Portal Use in Orthopaedic Surgery Is Associated with Disparities, Improved Satisfaction, and Lower No-Show Rates. *Journal of Bone & Joint Surgery, American Volume*. 2020;102(15):1336-1343. doi:10.2106/JBJS.19.01080

13. Witt E, Eruchalu C, Dey T, Bates D, Goodwin C, Ortega G. The role of patient primary language in access to brain tumor resection: Evaluating emergent admission and hospital volume. *JOURNAL OF CANCER POLICY*. 2021;30. doi:10.1016/j.jcpo.2021.100306

14. Maurer LR, Allar BG, Perez NP, et al. Non-English Primary Language is Associated with Emergency Surgery for Diverticulitis. *Journal of Surgical Research*. 2021;268:643-649. doi:10.1016/j.jss.2021.07.042

15. Nashed M, Glantz MJ, Linskey ME, Bota DA. Access to specialized treatment by adult Hispanic brain tumor patients: findings from a single-institution retrospective study. *Community Oncology*. 2012;9(9):283-288.

16. Thompson A, Ivey S, Lahiff M, Betjemann J. Delays in time to surgery for minorities with temporal lobe epilepsy. *EPILEPSIA*. 2014;55(9):1339-1346. doi:10.1111/epi.12700

17. Jaiswal K, Hull M, Furniss AL, Doyle R, Gayou N, Bayliss E. Delays in Diagnosis and Treatment of Breast Cancer: A Safety-Net Population Profile. *J Natl Compr Canc Netw*. 2018;16(12):1451-1457. doi:10.6004/jnccn.2018.7067

18. John-Baptiste A, Naglie G, Tomlinson G, et al. The effect of English language proficiency on length of stay and in-hospital mortality. *J Gen Intern Med*. 2004;19(3):221-228. doi:10.1111/j.1525-1497.2004.21205.x

19. Tang EW, Go J, Kwok A, et al. The relationship between language proficiency and surgical length of stay following cardiac bypass surgery. *European Journal of Cardiovascular Nursing*. 2016;15(6):438-446. doi:10.1177/1474515115596645

20. Inagaki E, Farber A, Kalish J, et al. Role of language discordance in complication and readmission rate after infrainguinal bypass. *Journal of Vascular Surgery*. 2017;66(5):1473-1478. doi:10.1016/j.jvs.2017.03.453

21. Hyun KK, Redfern J, Woodward M, et al. Is There Inequity in Hospital Care Among Patients With Acute Coronary Syndrome Who Are Proficient and Not Proficient in English Language? Analysis of the SNAPSHOT ACS Study. *Journal of Cardiovascular Nursing*. 2017;32(3):288-295. doi:10.1097/JCN.0000000000000342

22. Feeney T, Cassidy M, Tripodis Y, et al. Association of Primary Language with Outcomes After Operations Typically Performed to Treat Cancer: Analysis of a Statewide Database. *Ann Surg Oncol*. 2019;26(9):2684-2693. doi:10.1245/s10434-019-07484-8

23. Feeney T, Sanchez S, Tripodis Y, et al. The Association of Primary Language With Emergency General Surgery Outcomes Using a Statewide Database. *JOURNAL OF SURGICAL RESEARCH*. 2019;244:484-491. doi:10.1016/j.jss.2019.06.082

24. Feeney T, Park C, Godley F, et al. Provider–patient Language Discordance and Cancer Operations: Outcomes from a Single Center Linked to a State Vital Statistics Registry. *World J Surg*. 2020;44(10):3324-3332. doi:10.1007/s00268-020-05614-y

25. Bernstein JA, Sharan M, Lygrisse KA, Lajam CM. The Need for an Interpreter Increases Length of Stay and Influences Postoperative Disposition Following Primary Total Joint Arthroplasty. *Journal of Arthroplasty*. 2020;35(9):2405-2409. doi:10.1016/j.arth.2020.04.083

26. Witt EE, Eruchalu CN, Dey T, Bates DW, Goodwin CR, Ortega G. Non-English Primary Language Is Associated with Short-Term Outcomes After Supratentorial Tumor Resection. *World Neurosurgery*. 2021;155:e484-e502. doi:10.1016/j.wneu.2021.08.087

27. Manuel SP, Nguyen K, Karliner LS, Ward DT, Fernandez A. Association of English Language Proficiency With Hospitalization Cost, Length of Stay, Disposition Location, and Readmission Following Total Joint Arthroplasty. *JAMA Network Open*. 2022;5(3):e221842-e221842. doi:10.1001/jamanetworkopen.2022.1842

28. Manuel SP, Chia ZK, Raygor KP, Fernández A. Association of Language Barriers With Process Outcomes After Craniotomy for Brain Tumor. *Neurosurgery*. 2022;91(4):590-595. doi:10.1227/neu.0000000000002080

29. Stolarski AE, Alonso A, Aly S, et al. The impact of English proficiency on outcomes after bariatric surgery. *Surg Endosc*. 2022;36(10):7385-7391. doi:10.1007/s00464-022-09148-3

30. Kovoor J, Bacchi S, Stretton B, et al. Language and gender barriers to pain control after general surgery. *ANZ JOURNAL OF SURGERY*. doi:10.1111/ans.18164

31. Schwartz HEM, Matthay ZA, Menza R, et al. Inequity in discharge pain management for trauma patients with limited English proficiency. *Journal of Trauma & Acute Care Surgery*. 2021;91(5):898-902. doi:10.1097/TA.0000000000003294

32. Dowsey MM, Broadhead ML, Stoney JD, Choong PF. Outcomes of Total Knee Arthroplasty in English-Versus Non–English-Speaking Patients. *J Orthop Surg (Hong Kong)*. 2009;17(3):305-309. doi:10.1177/230949900901700312

33. Wong D, Roth E, Sokas C, et al. Preventable Emergency Department Visits After Colorectal Surgery. *DISEASES OF THE COLON & RECTUM*. 2021;64(11):1417-1425. doi:10.1097/DCR.0000000000002127

34. Danilowicz DA, Gabriel HP. Postcardiotomy psychosis in non-English-speaking patients. *Psychiatry Med*. 1971;2(4):314-320. doi:10.2190/3LRQ-NR4X-BFHM-0PYW

35. Dzioba RB, Doxey NC. A prospective investigation into the orthopaedic and psychologic predictors of outcome of first lumbar surgery following industrial injury. *Spine (Phila Pa 1976)*. 1984;9(6):614-623. doi:10.1097/00007632-198409000-00013

© 2023 Joo H et al. *JAMA Network Open.*

36. Doxey NC, Dzioba RB, Mitson GL, Lacroix JM. Predictors of outcome in back surgery candidates. *J Clin Psychol*. 1988;44(4):611-622. doi:10.1002/1097-4679(198807)44:4<611::aid-jclp2270440419>3.0.co;2-g

37. NAYLOR G. IRIS PROLAPSE - WHO - WHEN - WHY. *EYE*. 1993;7:465-467. doi:10.1038/eye.1993.94

38. John-Baptiste A, Naglie G, Tomlinson G, et al. The effect of English language proficiency on length of stay and in-hospital mortality. *J Gen Intern Med*. 2004;19(3):221-228. doi:10.1111/j.1525-1497.2004.21205.x

39. Ernest CS, Elliott PC, Murphy BM, et al. Predictors of cognitive function in candidates for coronary artery bypass graft surgery. *J Int Neuropsychol Soc*. 2007;13(2):257-266. doi:10.1017/S1355617707070282

40. Bandyopadhyay M, Markovic M, Manderson L. Women's perspectives of pain following day surgery in Australia. *Aust J Adv Nurs*. 2007;24(4):19-23.

41. Clapp B, Jarmillo M, Vigil V, et al. Patient comprehension and recall of laparoscopic surgery and outcomes in a non-English speaking population. *JSLS*. 2007;11(2):242-245.

42. Hawley ST, Janz NK, Hamilton A, et al. Latina patient perspectives about informed treatment decision making for breast cancer. *Patient Educ Couns*. 2008;73(2):363-370. doi:10.1016/j.pec.2008.07.036

43. Maly R, Liu Y, Kwong E, Thind A, Diamant A. Breast Reconstructive Surgery in Medically Underserved Women With Breast Cancer The Role of Patient-Physician Communication. *CANCER*. 2009;115(20):4819-4827. doi:10.1002/cncr.24510

44. Dowsey MM, Broadhead ML, Stoney JD, Choong PF. Outcomes of Total Knee Arthroplasty in English-Versus Non–English-Speaking Patients. *J Orthop Surg (Hong Kong)*. 2009;17(3):305-309. doi:10.1177/230949900901700312

45. Halpern MT, Holden DJ. Patient and health system disparities in timeliness of treatment for individuals with colorectal cancer (CRC). *Journal of Clinical Oncology*. 2009;27:6540-6540.

46. Nielsen SS, He Y, Ayanian JZ, et al. Quality of cancer care among foreign-born and US-born patients with lung or colorectal cancer. *Cancer (0008543X)*. 2010;116(23):5497-5506. doi:10.1002/cncr.25546

47. Clark S, Mangram A, Ernest D, Lebron R, Peralta L. The Informed Consent: A Study of the Efficacy of Informed Consents and the Associated Role of Language Barriers. *JOURNAL OF SURGICAL EDUCATION*. 2011;68(2):143-147. doi:10.1016/j.jsurg.2010.09.009

48. Campesino M, Koithan M, Ruiz E, et al. Surgical treatment differences among Latina and African American breast cancer survivors. *Oncol Nurs Forum*. 2012;39(4):E324-331. doi:10.1188/12.ONF.E324-E331

49. Alnaes AH. Lost in translation: cultural obstructions impede living kidney donation among minority ethnic patients. *Camb Q Healthc Ethics*. 2012;21(4):505-516. doi:10.1017/S0963180112000278

© 2023 Joo H et al. *JAMA Network Open.*

50. Betjemann J, Thompson A, Santos-Sanchez C, Garcia P, Ivey S. Distinguishing language and race disparities in epilepsy surgery. *EPILEPSY & BEHAVIOR*. 2013;28(3):444-449. doi:10.1016/j.yebeh.2013.06.020

51. Dowsey M, Nikpour M, Choong P. Outcomes following large joint arthroplasty: does socio-economic status matter? *BMC MUSCULOSKELETAL DISORDERS*. 2014;15. doi:10.1186/1471-2474-15-148

52. Ankuda CK, Block SD, Cooper Z, et al. Measuring critical deficits in shared decision making before elective surgery. *Patient Education & Counseling*. 2014;94(3):328-333. doi:10.1016/j.pec.2013.11.013

53. Lopez M, Kaplan C, Napoles A, Hwang E, Livaudais J, Karliner L. Satisfaction with treatment decision-making and treatment regret among Latinas and non-Latina whites with DCIS. *PATIENT EDUCATION AND COUNSELING*. 2014;94(1):83-89. doi:10.1016/j.pec.2013.09.005

54. Alley MC, Mason AS, Tybor DJ, Pevear ME, Baratz MD, Smith EL. Ethnic Barriers to Utilization of Total Joint Arthroplasty Among Chinese Immigrants in the United States. *Journal of Arthroplasty*. 2016;31(9):1873-1877.e2. doi:10.1016/j.arth.2016.02.046

55. Patel DN, Wakeam E, Genoff M, Mujawar I, Ashley SW, Diamond LC. Preoperative consent for patients with limited English proficiency. *Journal of Surgical Research*. 2016;200(2):514-522. doi:10.1016/j.jss.2015.09.033

56. Inagaki E, Zhu CK, Farber A, et al. Role of language discordance in readmission rate and complications after infrainguinal bypass. *J Vasc Surg*. 2016;63(6):181S. doi:10.1016/j.jvs.2016.03.306

57. Spence J, Bosch J, Sharma M, et al. Predictors of cognitive decline after cardiac surgery: An evaluation of the CABG off or on pump revascularization study (CORONARY) cohort. *Can J Cardiol*. 2017;33(10):S36. doi:10.1016/j.cjca.2017.07.089

58. Talamantes E, Norris KC, Mangione CM, et al. Linguistic Isolation and Access to the Active Kidney Transplant Waiting List in the United States. *Clin J Am Soc Nephrol*. 2017;12(3):483-492. doi:10.2215/CJN.07150716

59. Jaiswal K, Hull M, Furniss A, Doyle R, Gayou N, Bayliss E. Delays in Diagnosis and Treatment of Breast Cancer: A Safety-Net Population Profile. *JOURNAL OF THE NATIONAL COMPREHENSIVE CANCER NETWORK*. 2018;16(12):1451-1457. doi:10.6004/jnccn.2018.7067

60. Yoo F, Ference EH, Kuan EC, Lee JT, Wang MB, Suh JD. Evaluation of patient nasal saline irrigation practices following endoscopic sinus surgery. *Int Forum Allergy Rhinol*. 2018;8(1):32-40. doi:10.1002/alr.22034

61. Talutis SD, Kuhnen AH, Zhu C, Pearlman K, Hall J. Effect of primary language on readmission after colorectal surgery. *Dis Colon Rectum*. 2018;61(5):e166-e167. doi:10.1097/DCR.0000000000001104

© 2023 Joo H et al. *JAMA Network Open.*

62. Shiraev T, Durur E, Robinson D. Factors Predicting Noncompliance with Follow-up after Endovascular Aneurysm Repair. *ANNALS OF VASCULAR SURGERY*. 2018;52:30-35. doi:10.1016/j.avsg.2018.03.037

63. Patel A, Wang W, Warnack E, et al. Surgical treatment of young women with breast cancer: Public vs private hospitals. *BREAST JOURNAL*. 2019;25(4):625-630. doi:10.1111/tbj.13294

64. Feeney T, Cassidy M, Tripodis Y, et al. Association of Primary Language with Outcomes After Operations Typically Performed to Treat Cancer: Analysis of a Statewide Database. *ANNALS OF SURGICAL ONCOLOGY*. 2019;26(9):2684-2693. doi:10.1245/s10434-019-07484-8

65. Rosenbloom JM, Jackson J, Alegria M, Alvarez K. Healthcare provider perceptions of disparities in perioperative care. *J Natl Med Assoc*. 2019;111(6):616-624. doi:10.1016/j.jnma.2019.07.003

66. Schultz KS, Phatak UR, Hall JF, Favuzza J. Impact of Insurance Status on Clinical Outcomes after Colorectal Surgery at a Safety-Net Hospital. *J Am Coll Surg*. 2020;231(4):e101. doi:10.1016/j.jamcollsurg.2020.08.257

67. Cataneo JL, Minhem M, M O'Rear J, et al. Socioeconomic Status and English Language Proficiency Alter Outcomes after Appendectomy. *Journal of the American College of Surgeons*. 2020;231(4):e138-e139. doi:10.1016/j.jamcollsurg.2020.08.364

68. Hong J, De Roulet A, Chao S. Equity in Postoperative Outcomes in Geriatric Patients Requiring Non-elective Colectomy in a High-density, Multilingual Hospital Setting. *J Am Coll Surg*. 2021;233(5):e29. doi:10.1016/j.jamcollsurg.2021.08.081

69. Burgoon ML, Miller PA, Hoover-Hankerson B, Strand N, Ross H. Challenges to Understanding and Compliance Among Surgical Patients in Low-Income Urban Teaching Hospitals. *Am Surg*. 2021;87(5):818-824. doi:10.1177/0003134820960078

70. Witt EE, Eruchalu CN, Dey T, Bates DW, Goodwin CR, Ortega G. Non-English Primary Language Is Associated with Short-Term Outcomes After Supratentorial Tumor Resection. *World Neurosurgery*. 2021;155:e484-e502. doi:10.1016/j.wneu.2021.08.087

71. Greenberg A, Schwartz H, Collins C, et al. Emergency general surgery utilization and disparities during COVID-19: an interrupted time-series analysis. *TRAUMA SURGERY & ACUTE CARE OPEN*. 2021;6(1). doi:10.1136/tsaco-2021-000679

72. Shehan JN, Alwani T, LeClair J, et al. Social determinants of health and treatment decisions in head and neck cancer. *Head Neck*. 2022;44(2):372-381. doi:10.1002/hed.26931

73. Tang A, Mittal A, Mooney C, et al. Factors delaying chemotherapy in patients with breast cancer at a safety-net hospital. *JOURNAL OF THE NATIONAL MEDICAL ASSOCIATION*. 2022;113(6):706-712. doi:10.1016/j.jnma.2021.08.035

74. de Crescenzo C, Chen YW, Adler J, et al. Increasing Frequency of Interpreting Services is Associated With Shorter Peri-operative Length of Stay. *J Surg Res*. 2022;270((de Crescenzo C., cdecrescenzo@mgh.harvard.edu; Chen Y.-W.; Maurer L.R.; Chang D.C.;

Yeh H.) Department of Transplant Surgery, Massachusetts General Hospital/Harvard Medical School, Boston, MA, United States):178-186. doi:10.1016/j.jss.2021.09.006

75. Norris AC, Mears SC, Siegel ER, Barnes CL, Stambough JB. Social Needs of Patients Undergoing Total Joint Arthroplasty. *J Arthroplasty*. 2022;37(7S):S416-S421. doi:10.1016/j.arth.2021.11.019

76. Khan F, Soodana-Prakash N, Martin L, Syan R, Amin K. Language-Related Disparities in Acute Postoperative Pain Management Following Pelvic Floor Reconstructive Surgery: Does English Proficiency Matter? *INTERNATIONAL UROGYNECOLOGY JOURNAL*. 2022;33(SUPPL 2):S516-S517.

77. Dirix M, Philipse E, Vleut R, et al. Timing of the pre-transplant workup for renal transplantation: is there room for improvement? *CLINICAL KIDNEY JOURNAL*. doi:10.1093/ckj/sfac006

78. Barnard J, Milne T, Teo K, Weston M, Israel L, Peng S. Causes and costs of delayed closure of ileostomies in rectal cancer patients in Australasian units. *ANZ JOURNAL OF SURGERY*. doi:10.1111/ans.18092

79. Plocienniczak M, Rubin BR, Kolli A, Levi J, Tracy L. Outcome Disparities and Resource Utilization Among Limited English Proficient Patients After Tonsillectomy. *Ann Otol Rhinol Laryngol*. 2022;131(11):1241-1246. doi:10.1177/00034894211061996

80. Alwani T, Shehan JN, LeClair J, et al. Geographic Barriers Affect Follow-Up Care in Head and Neck Cancer. *Laryngoscope*. 2022;132(5):1022-1028. doi:10.1002/lary.29934

81. Jimenez N, Jackson DL, Zhou C, Ayala NC, Ebel BE. Postoperative pain management in children, parental english profi ciency, and access to interpretation. *Hosp Pediatr*. 2014;4(1):23-30. doi:10.1542/hpeds.2013-0031

82. Dai X, Ryan MA, Clements AC, et al. The Effect of Language Barriers at Discharge on Pediatric Adenotonsillectomy Outcomes and Healthcare Contact. *Annals of Otology, Rhinology & Laryngology*. 2021;130(7):833-839. doi:10.1177/0003489420980176

83. Essex R, Cucos M, Dibley L. The impact of language and ethnicity on preparation for endoscopy: A prospective audit of an East London Hospital Ward. *J Eval Clin Pract*. 2021;27(4):877-884. doi:10.1111/jep.13490

84. Greene NEB, Fuentes-Juarez BN, Sabatini CSM MPH, Greene NE, Fuentes-Juárez BN, Sabatini CS. Access to Orthopaedic Care for Spanish-Speaking Patients in California. *Journal of Bone & Joint Surgery, American Volume*. 2019;101(18):e95-e95. doi:10.2106/JBJS.18.01080

85. Jaramillo J, Snyder E, Dunlap J, Wright R, Mendoza F, Bruzoni M. The Hispanic Clinic for Pediatric Surgery: A model to improve parent-provider communication for Hispanic pediatric surgery patients. *JOURNAL OF PEDIATRIC SURGERY*. 2016;51(4):670-674. doi:10.1016/j.jpedsurg.2015.08.065

86. Lee J, Pérez-Stable E, Gregorich S, et al. Increased Access to Professional Interpreters in the Hospital Improves Informed Consent for Patients with Limited English Proficiency. *JGIM: Journal of General Internal Medicine*. 2017;32(8):863-870. doi:10.1007/s11606-017-3983-4

87. Malevanchik L, Wheeler M, Gagliardi K, Karliner L, Shah SJ. Disparities After Discharge: The Association of Limited English Proficiency and Postdischarge Patient-Reported Issues. *Jt Comm J Qual Patient Saf*. 2021;47(12):775-782. doi:10.1016/j.jcjq.2021.08.013

88. Plancarte CA, Hametz P, Southern WN. Association Between English Proficiency and Timing of Analgesia Administration After Surgery. *Hospital Pediatrics*. 2021;11(11):1199-1204. doi:10.1542/hpeds.2020-005766

89. Qureshi M, Romesser P, Jalisi S, et al. The influence of limited English proficiency on outcome in patients treated with radiotherapy for head and neck cancer. *PATIENT EDUCATION AND COUNSELING*. 2014;97(2):276-282. doi:10.1016/j.pec.2014.07.031

© 2023 Joo H et al. *JAMA Network Open.*

Supplementary Tables

eTable 1. Study Outcomes, Methods, and Results by Category

**Source**	**Definition of Outcome**	**Statistical Methodology**	**Adjusted Covariates**	**Measure of Association**	**Magnitude and Direction of Associations Between LEP (vs. EP) and Outcome Measures**	**Significant Directionality**
**Access to Surgical Care**						
Receipt of Surgery						
Sridhar et al,[9] 2019	Receipt of curative pancreatic cancer surgery	Pearson's chi-square test	None	Independence between LEP status and receipt of surgery	No association between LEP and receipt of surgery (p-value = 0.79)	.
Asokan et al,[10] 2020	Receipt of esophagectomy in patients with operable stage	Pearson's chi-square test	None	Independence between LEP status and receipt of surgery	LEP and receipt of surgery were independent (p-value = 0.103)	.
Betjemann et al,[11] 2020	Underwent anterior temporal lobectomy after screening confirmed refractory epilepsy and mesial temporal sclerosis	Multivariable logistic regression model	Age, race/ethnicity, ictal EEG	Adjusted OR for use in surgery (LEP/EP)	Reduced surgery utilization in LEP, aOR 0.38 (0.15-0.93)	↓
Availability of High-quality Surgical Care						

© 2023 Joo H et al. *JAMA Network Open.*

Varady et al,[12] 2020	Use of electronic patient portal (EPP) by the time of orthopedic surgical procedure, defined as having completed 1> online sessions	Multivariable logistic regression model	Age, sex, race, medications, education level, insurance type, income, distance to hospital, provider subspecialty, primary care physician	Adjusted OR for EPP utilization (LEP/EP)	Reduced use among LEP: aOR, 0.42 (0.36-0.50)	↓
Witt et al,[13] 2021	Total number of supratentorial tumor resections per year (higher vs. lower volume)	Multivariable ordinal logistic regression	Age, sex, diagnosis, insurance status, household income, year of discharge, residence, Chalrson comorbidity score	Adjusted OR for higher hospital volume (Spanish/EP OR NENS/EP)	LEP (Spanish) vs. EP, aOR 0.84 (0.67-1.05); LEP (NENS) vs. EP, aOR 1.07 (0.85-1.35)	.
Elective vs. Emergency surgery						
Witt et al,[13] 2021	Routine admission vs. emergent or urgent admission for neuro-oncologic surgery	Multivariable logistic regression model	Age, sex, diagnosis, insurance status, household income, year of discharge, residence, Chalrson comorbidity score	Adjusted OR for emergent/urgent admission (Spanish/EP OR NENS/EP	LEP (Spanish) vs. EP, aOR 1.02 (0.78-1.33); LEP (NENS) vs. EP, aOR 0.48 (0.34-0.67)	.↓
Maurer et al,[14] 2021	Elective vs. emergent or urgent admission for colectomy	Multivariable logistic regression model	Age, sex, insurance status, income, data year, Charlson comorbidity index	Adjusted OR for limited access (LEP/EP)	LEP associated decreased odds of elective surgery: aOR 0.74 (0.62-0.88)	↓

© 2023 Joo H et al. *JAMA Network Open.*

**Delay in Surgical Care**						
Nashed et al,[15] 2012	Time from diagnosis to craniotomy	Log rank test of equality	None	Difference in time-to-event between LEP & EP	No difference in time to surgery (p-value=0.26)	.
Thompson et al,[16] 2014	Time from presurgical evaluation to anterior temporal lobectomy	Log rank test of equality	None	Difference in time-to event between LEP & EP	LEP associated with longer time to surgery than EP (p-value=0.0085)	↑
Jaiswal et al,[17] 2018	Time from breast cancer diagnosis to initial treatment (delayd treatment ≥37 days; timely treatment <37 days)	Simple logistic regression model	None	Unadjusted OR for delayed treatment (LEP/EP)	LEP associated with longer time to treatment: OR, 5.0 (95% CI N/S; p-value=0.0045)	↑
Silverstein et al,[8] 2022	Time from date of referral to first appointment with a MIGS provider (delayed interval > 30 days; timely interval < 30 days)	Multivariable logistic regression model	Referral indication (chronic pelvic pain vs. diagnosis that require operative intervention)	Adjusted OR for delayed interval (LEP/EP)	Delay in surgical care for LEP patients: pandemic cohort, aOR 3.20 (1.60-6.40); historic cohort, aOR 1.16 (0.55-2.41)	↑
**Length of Stay**						

© 2023 Joo H et al. *JAMA Network Open.*

John-Baptiste et al,[18] 2004	LOS during surgical admission	Multivariable log-linear regression model	Age, gender, discharge disposition, fiscal year, Charlson comorbidity score, number of comorbidities, marital status, income	Adjusted means ratio of LOS (LEP/EP)	Increased or no difference in LOS: CABG, aMR 1.07 (1.03-1.12); Prostectomy, aMR 1.02 (0.93-1.11); Craniotomy, aMR 1.15 (1.02-1.31); Hysterectomy, aMR 0.97 (0.92-1.02); Intestinal & rectal, aMR 1.10 (1.02-1.19); Elective hip replacement, aMR 1.13 (1.03-1.23); Hip fracture, aMR 0.98 (0.88-1.09); Head & neck, aMR 0.93 (0.73-1.19); Elective AAA repair, aMR 1.00 (0.84-1.20); AAA rupture, aMR 1.46 (0.48-4.45)	.↑
MacDonald et al,[5] 2010	LOS (long LOS, 7>days; short LOS, 6<days) after hip and knee arthroplasty	Multivariable logistic regression model	Age, comorbidities, required home support, living alone	Adjusted OR for long LOS (LEP/EP)	LEP patients had prolonged LOS: aOR 4.15 (95% CI N/S; p-value <0.05)	↑
Tang et al,[19] 2016	LOS defined as time from CSICU admission to hospital discharge after CABG. LOS was categorized into quartiles (1-5 days, 6 days, 7-8 days, and >9 days)	Multivariate polynomial regression model	Age, marital status, postoperative infection	Adjusted OR for each quartile of LOS (LEP/EP)	No difference in LOS: 6 days vs 1-5 days aOR 1.52 (0.77-2.98); 7-8 days vs. 1-5 days aOR 1.55 (0.81-2.97); >9 days vs 1-5 days aOR 1.85 (0.94-3.67)	.

© 2023 Joo H et al. *JAMA Network Open.*

Inagaki et al,[20] 2017	Postoperative LOS after infrainguinal bypass surgery	Multivariable gamma regression model	Age, gender, race, ethnicity, insurance status, tobacco use, CAD, CHF, COPD, cerebrovascular accident, renal failure, DM, urgency of care, outflow artery, graft type	Adjusted means ratio of LOS (LEP/EP)	No difference in LOS: aMR 1.02 (0.85-1.23)	.
Hyun et al,[21] 2017	LOS in days after admission with suspected ACS event	Multiple linear regression model	Gender, GRACE risk score, previous diagnosis and procedures, presenting diagnosis, medications, PCI, CABG	Difference in mean LOS between LEP & EP	No difference in LOS between LEP and EP: Effect size N/S (p-value =0.30)	.

© 2023 Joo H et al. *JAMA Network Open.*

Feeney et al,[22] 2019	LOS in days after oncologic surgery	Generalized linear mixed model with negative binomial distribution	Age, gender, race, insurance type, income, DM, obesity, psychiatric illness, alcohol abuse, HTN, cancer with metastasis, chronic lung disease, peripheral vascular disease, renal failure, number of comorbidities/procedures, risk class, country of residence, cancer, weekend admission, hospital	Adjusted IRR for LOS (Spanish/EP OR NENS/EP)	No difference in LOS: LEP (Spanish) vs. EP, aIRR 1.02 (0.98-1.06); LEP (NENS) vs. EP, aIRR 1.03 (0.99-1.07)	.
Feeney et al,[23] 2019	LOS during hospitalization for emergency surgery	Generalized linear mixed model with negative binomial distribution	Age, sex, race, insurance type, income, comorbidities, number of hospital procedures, risk class, country of residence, cancer, weekend admission, admission hour	Adjusted IRR for LOS (Spanish/EP OR NENS/EP)	Spanish speakers had reduced LOS after appendectomy: LEP (Spanish) vs. EP, aIRR 0.92 (0.89-0.95), LEP (NENS) vs. EP, aIRR 0.96 (0.92-1.00); cholecystectomy, LEP (Spanish) vs. EP, aIRR 1.02 (0.99-1.04), LEP (NENS) vs. EP, aIRR 1.03 (0.99-1.07); Spanish speakers had reduced LOS after adhesiolysis: LEP (Spanish) vs. EP, aIRR 0.93 (0.88-0.97), LEP (NENS) vs. EP, aIRR 0.94	Mixed

© 2023 Joo H et al. *JAMA Network Open.*

					(0.89-0.99); Spanish speakers had longer LOS after high-risk procedures: LEP (Spanish) vs. EP, aIRR 1.14 (1.10-1.20), LEP (NENS) vs. EP, aIRR 1.04 (1.00-1.09)	
Feeney et al,[24] 2020	LOS in days	Multivariable negative binomial regression model	Age, gender, race/ethnicity, risk score, insurance status, income, BMI, emergency surgery, Elixhauser comorbidity score, ASA class, and weekend admission	Adjusted IRR for LOS (LEP/EP)	No difference in LOS: aIRR 0.99 (0.88-1.10)	.
Bernstein, et al,[25] 2020	LOS in days after primary total joint arthroplasty	Multivariable linear regression model	Age, ASA status	Difference in mean LOS between LEP & EP	Longer LOS for LEP-I or LEP-N than EP (2.72 or 2.44 vs. 2.19 days; p-value <0.0001 and p-value=0.012, respectively)	↑

© 2023 Joo H et al. *JAMA Network Open.*

Witt et al,[26] 2021	Total LOS after neuro-oncologic surgery<br><br>Postoperative LOS after neuro-oncologic surgery	Multivariable negative binomial regression model	Age, sex, insurance status, income, Charlson Comorbidity Index, year of discharge, weekend admission, emergency admission, hospital admission, hospital volume, inpatient complication, total number of procedures, discharge disposition	Adjusted IRR for LOS (Spanish/EP OR NENS/EP)<br><br>Adjusted IRR for postoperative LOS (Spanish/EP OR NENS/EP)	No difference in LOS: LEP (Spanish) vs. EP, aIRR 1.02 (0.96-1.09); LEP (NENS) vs. EP, aIRR 1.04 (0.98-1.11)<br><br>Longer postop LOS for NENS: LEP (Spanish) vs. EP, aIRR 1.01 (0.92-1.10); LEP (NENS) vs. EP, aIRR 1.10 (1.03-1.18)	.<br><br>.↑
Manuel et al,[27] 2022	Total LOS after total knee and hip arthroplasty	Multivariable negative binomial regression model	Age, sex, race/ethnicity, insurance type, ASA status, BMI, surgical case class, case length, estimated blood loss, discharge disposition	Adjusted IRR for LOS (LEP/EP)	Increased LOS for LEP: aIRR 1.15 (1.07-1.25)	↑
Manuel et al,[28] 2022	Total LOS after craniotomy	Multivariable negative binomial regression model	Age, sex, race/ethnicity, insurance type, ASA status, surgical case class, discharge disposition	Adjusted IRR for LOS (LEP/EP)	Increased LOS for LEP: aIRR 1.11 (1.00-1.24)	↑

© 2023 Joo H et al. *JAMA Network Open.*

Stolarski et al,[29] 2022	LOS from index operation (laparoscopic sleeve gastrectomy or gastric bypass)	Multivariable negative binomial regression model	Age, sex, race, insurance status, ASA class, smoking status, year of operation, procedure type	Adjusted IRR for LOS (LEP/EP)	No difference in LOS: aIRR 0.94 (0.84-1.04)	.
Kovoor, et al,[30] 2022	LOS dichotomized at 75th percentile (>5 days) for general surgery admission	Multivariable logistic regression model	Age, gender, marital status, pain scores, in-hospital mortality, birth country, religion, SES, Charlson Comorbidity Index, time of admission	Adjusted OR for LOS $\geq$5 days (LEP/EP)	No difference in LOS: aOR, 1.08 (0.94-1.25)	.
**Discharge Disposition**						
Bernstein, et al,[25] 2020	Discharge disposition after primary total joint arthroplasty	Multivariable linear regression model	Age, ASA status	Difference in discharge disposition between LEP & EP	Increased disposition to skilled nursing for LEP-I than EP (25.3% vs 9.3%; p-value <0.0001); no difference in disposition to skilled nursing between LEP-N and EP (14.0% vs. 9.3%; p-value=0.144)	.↑
Witt et al,[26] 2021	Discharge disposition to rehabilitation (vs. home) after neuro-oncologic surgery	Multivariable logistic regression model	Age, sex, insurance status, income, Charlson Comorbidity Index, year of discharge, weekend admission, emergency	Adjusted OR for rehabilitation discharge (LEP/EP)	Decreased or no difference in disposition to skilled nursing for LEP than EP: LEP (Spanish) vs. EP, aOR 0.65(0.45-0.93); LEP (NENS) vs. EP, aOR 1.00 (0.80-1.25)	.↓

© 2023 Joo H et al. *JAMA Network Open.*

			admission, hospital volume			
Manuel et al,[27] 2022	Discharge disposition to skilled facility (vs. home) after total knee or hip arthroplasty	Multivariable logistic regression model	Age, sex, race/ethnicity, insurance type, ASA status, BMI, surgical case class, case length, and estimated blood loss	Adjusted OR for skilled facility discharge (LEP/EP)	Increased discharge to skilled facility for LEP: aOR 1.41 (1.03-1.93)	↑
Manuel et al,[28] 2022	Discharge disposition to skilled facility (vs. home) after craniotomy	Multivariable logistic regression model	Age, sex, race/ethnicity, insurance type, ASA status, and surgical case class	Adjusted OR for skilled facility discharge (LEP/EP)	Increased discharge to skilled facility for LEP: aOR 1.76 (1.13-2.72)	↑
**In-hospital Mortality**						
John-Baptiste et al,[18] 2004	In-hospital death during surgical admission	Multivariable logistic regression model	Age, Charlson comorbidity score	Adjusted OR for death (LEP/EP)	Increased or no difference in mortality: CABG, aOR 1.43 (0.97-2.11); Craniotomy, aOR 1.98 (1.34-2.94); Intestinal & rectal, aOR 0.60 (0.30-1.19); Hip fracture, aOR 0.66 (0.33-1.30); AAA rupture, aOR 7.34 (1.65-32.67)	.↑

© 2023 Joo H et al. *JAMA Network Open.*

Hyun et al,[21] 2017	In-hospital death during admission for suspected ACS event	Multivariable logistic regression model	Gender, GRACE risk score, previous cardiac diagnosis and procedures, presenting diagnosis	Adjusted OR for in-hospital death (LEP/EP)	No difference in mortality: aOR 1.77 (0.90-3.53)	.
Feeney et al,[22] 2019	In-hospital all-cause mortality after oncologic surgery	Generalized linear mixed model with Bernoulli distribution	Age, gender, race, insurance type, income, DM, obesity, psychiatric illness, alcohol abuse, HTN, cancer with metastasis, chronic lung disease, peripheral vascular disease, renal failure, number of comorbidities/procedures, LOS, risk class, country of residence, cancer, hospital	Adjusted OR for in-hospital death (Spanish/EP OR NENS/EP)	No difference in mortality: LEP (Spanish) vs. EP, aOR 0.67 (0.41-1.10); LEP (NENS) vs. EP, aOR 1.16 (0.77-1.75)	.
Feeney et al,[23] 2019	In-hospital all-cause mortality after emergency surgery	Generalized linear mixed model with Bernoulli distribution	Age, gender, race, insurance type, income, Elixhauser comorbidity score, number of comorbidities/procedures, LOS, risk class, country of residence, cancer	Adjusted OR for in-hospital death (Spanish/EP OR NENS/EP)	No difference in mortality: Appendectomy, LEP (Spanish) vs. EP, aOR 0.61 (0.14-2.60), LEP (NENS) vs. EP, aOR 0.84 (0.19-3.60); cholecystectomy, LEP (Spanish) vs. EP, aOR 0.41 (0.13-1.33), LEP (NENS) vs. EP, aOR 0.51 (0.18-1.43); adhesiolysis, LEP (Spanish) vs. EP, aOR 0.92 (0.43-2.00), LEP	.

					(NENS) vs. EP, aOR 0.53 (0.23-1.22); high-risk procedures, LEP (Spanish) vs. EP, aOR 1.01 (0.68-1.50), aOR LEP (NENS) vs. EP, 0.89 (0.63-1.25)	
Witt et al,[26] 2021	In-hospital mortality after neuro-oncologic surgery	Pearson's chi-square test	None	Difference in in-hospital mortality across LEP statuses	No difference across LEP statuses in in-hospital mortality (p-value = 0.127)	.
Kovoor, et al,[30] 2022	In-hospital mortality	Multivariable logistic regression model	Age, gender, marital status, LOS, pain scores, birth country, religion, SES, Charlson Comorbidity Index, time of admission	Adjusted OR for in-hospital mortality (LEP/EP)	No difference in mortality (p-value = 0.17) and aOR not reported	.
**Complications**						

Inagaki et al,[20] 2017	30-day would infections, 30-day adverse graft event after infrainguinal bypass surgery	Multivariable logistic regression model	Age, gender, race, ethnicity, insurance status, tobacco use, CAD, CHF, COPD, cerebrovascular accident, renal failure, DM, urgency of care, outflow artery, graft type	Adjusted OR for 30-day wound infections (LEP/EP) Adjusted OR for 30-day adverse graft event (LEP/EP)	No difference in 30-day complications rate: wound infections, aOR1.87 (0.90-3.88); adverse graft event aOR 1.23 (0.62-2.45)	.
Hyun et al,[21] 2017	In-hospital MACE; Delayed <18 months MACE after admission for ACS event	Multivariable logistic regression model	Gender, GRACE risk score, previous cardiac diagnosis and procedures, presenting diagnosis, medications, PCI, CABG, referral to rehabilitation	Adjusted OR for in-hospital MACE (LEP/EP) Adjusted OR for 18-month MACE (LEP/EP)	No difference in complications rate: in-hospital MACE, aOR 1.19 (0.85-1.65); MACE ≤18 months aOR 1.01 (0.65-1.57)	.
Feeney et al,[24] 2020	In-hospital major complications after cancer surgery based on NSQIP risk calculator major morbidity definition	Multivariable logistic regression model	Age, insurance status, operative risk score, Elixhauser comorbidity score	Adjusted OR for major complication (LEP/EP)	No difference in LOS: aOR 0.76 (0.39-1.45)	.

Witt et al,[26] 2021	Presence of inpatient complications validated for supratentorial tumors	Multivariable logistic regression model	Age, sex, insurance status, income, Charlson Comorbidity Index, year of discharge, weekend admission, emergency admission, hospital volume	Adjusted OR for inpatient complication (Spanish/EP OR NENS/EP)	Increased complications in NENS patients: LEP (Spanish) vs. EP, 0.85 (0.63-1.15); LEP (NENS) vs. EP, 1.36 (1.06-1.75)	.↑
Stolarski et al,[29] 2022	Presence of 30-day complications after laparoscopic sleeve gastrectomy or gastric bypass	Multivariable logistic regression model	Age, sex, race, insurance status, ASA class, smoking status, year of operation, and procedure type	Adjusted OR for 30-day complications (LEP/EP)	No difference in complications: aOR 0.59 (0.32-1.05)	.
**Pain Management**						
Schwartz, et al,[31] 2021	Total oral morphine equivalent (OME) prescribed at discharge after admission to trauma surgery service<br><br>Total amount of opioids prescribed at discharge after admission to trauma surgery service	Multivariable logistic regression model<br><br>Multivariable quantile regression model	Age, injury severity, activation level, injury type, traumatic brain injury, limb fracture, and discharge service	Adjusted OR for receiving discharge opioids (LEP/EP)<br><br>Difference in mean total OME between LEP & EP	LEP less likely to receive discharge opioid prescription: aOR 0.61 (0.44-0.85)<br><br>LEP patients received 25.8 (-3.2-54.9) fewer OME than EP patients at 60th percentile; LEP patients received 45.0 (5.48-84.5) fewer OME than EP patients at 80th percentile	↓<br><br>↓

© 2023 Joo H et al. *JAMA Network Open.*

Kovoor, et al,[30] 2022	Having median pain score ≥3 during inpatient general surgical admission	Multivariable logistic regression model	Age, gender, marital status, LOS, in-hospital mortality, birth country, religion, SES, Charlson Comorbidity Index, time of admission	Adjusted OR for having higher pain (LEP/EP)	LEP associated with lower pain scores: aOR 0.61 (0.52-0.71)	↓
**Long-term Outcome**						
Dowsey, et al,[32] 2009	International Knee Society (IKS) score measure of function 12 months after TKA	Multivariable logistic regression model	Age, gender, birthplace, comorbid rheumatoid arthritis, obesity	Adjusted OR for IKS score >120 (LEP/EP)	LEP associated with higher odds of having >120 IKS scores at 12-month follow-up: aOR 0.36 (0.16-0.8)	↓
Aggarwal et al,[7] 2022	Patient-rated improvement 6 months after THA<br><br>Oxford hip score (OHS), measure of function and pain 6 months after THA	Multivariable logistic regression model<br><br>Multivariable linear regression model	Age, sex, education, BMI, previous THA, lower back pain, lower limb arthritis, depression/anxiety, comorbidities, ASA status, expected pain, expected function, preoperative EQ-VAS, preoperative OHS, JSN (or KL for OHS model), radiographic scores	Adjusted OR for improvement (LEP/EP)<br><br>Difference in mean OHS between LEP & EP	No difference in patient rated improvement: aOR 0.83 (0.49-1.40)<br><br>LEP patients scored worse on OHS: -1.95 (-3.18-0.72)	.<br><br>↓

Stolarski et al,[29] 2022	Excess weight loss at postoperative 1 year after laparoscopic sleeve gastrectomy or gastric bypass	Multivariable linear regression model	Age, sex, race, insurance status, ASA class, smoking status, year of operation, procedure type, BMI	Difference in mean EWL between LEP & EP	No difference in in EWL (regression coefficient, 95% CI, and p-value N/S)	.
**Readmission**						
Wilbur et al,[6] 2016	Readmission within 30 days after discharge from gynecologic oncology surgery admission	Mixed logistic regression	Age, race, BMI, insurance type, length of index admission LOS, comorbidities, ostomy, primary diagnosis, primary procedure, tobacco use, alcohol use, depression, social work screen, medications at discharge, income	Adjusted OR for 30-day readmission (LEP/EP)	LEP associated with readmission: aOR 3.36 (1.01-11.15)	↑
Inagaki et al,[20] 2017	Unplanned readmission within 30-days of discharge, emergency department (ED) visit within 30 days of discharge after infrainguinal bypass surgery	Multivariable logistic regression model	Age, gender, race, ethnicity, insurance status, tobacco use, CAD, CHF, COPD, cerebrovascular accident, renal failure, DM, urgency of care, outflow artery, graft type	Adjusted OR for 30-day readmission (LEP/EP) Adjusted OR for 30-day ED return visit (LEP/EP)	No difference in readmission, aOR 1.51 (0.77-2.95); ED return visit, aOR 1.28 (0.58-2.83)	.

© 2023 Joo H et al. *JAMA Network Open.*

Feeney et al,[22] 2019	7-day readmission after discharge from oncologic surgery	Generalized linear mixed model with Bernoulli distribution	Age, gender, race, insurance type, income, DM, obesity, psychiatric illness, alcohol abuse, HTN, cancer with metastasis, chronic lung disease, peripheral vascular disease, renal failure, number of comorbidities/procedures, risk class, country of residence, cancer, weekend admission, admission hour, and hospital	Adjusted OR for 7-day readmission (Spanish/EP OR NENS/EP)	No difference in readmission: LEP (Spanish) vs. EP, aOR 1.29 (0.93-1.80); LEP (NENS) vs. EP, aOR 0.80 (0.54-1.18)	.
Feeney et al,[23] 2019	7-day readmission after discharge from emergency surgery admission	Generalized linear mixed model with Bernoulli distribution	Age, gender, race, insurance status, income, Elixhauser comorbidity score, number of comorbidities/procedures, risk class, country of residence, cancer, weekend admission, admission hour	Adjusted OR for 7-day readmission (Spanish/EP OR NENS/EP)	No difference in readmission: Appendectomy, LEP (Spanish) vs. EP, aOR 0.65 (0.41-1.04), LEP (NENS) vs. EP, aOR 1.13 (0.63-2.03); cholecystectomy, LEP (Spanish) vs. EP, aOR 1.02 (0.74-1.42), LEP (NENS) vs. EP, aOR 0.85 (0.50-1.45); adhesiolysis, LEP (Spanish) vs. EP, aOR 0.73 (0.39-1.36), LEP (NENS) vs. EP, aOR 0.77	.

© 2023 Joo H et al. *JAMA Network Open.*

					(0.32-1.86); high-risk procedures, LEP (Spanish) vs. EP, aOR 1.45 (0.89-2.35), LEP (NENS) vs. EP, aOR 1.56 (0.94-2.59)	
Feeney et al,[24] 2020	30-day revisit to emergency department after discharge from cancer surgery	Multivariable logistic regression model	Age, gender, race/ethnicity, insurance status, risk score, income, BMI, emergency classification, Elixhauser comorbidity score, ASA class, LOS	Adjusted OR for 30-day revisit (LEP/EP)	No difference in 30-day revisits: aOR 1.08 (0.75-1.53)	.
Wong et al,[33] 2021	Postoperative emergency department visit within 30 days of discharge	Multinomial logistic regression	Age, race, gender, BMI, diagnosis, stoma, surgical approach, ASA status, frailty index, postoperative LOS, unexpected return to OR, discharge disposition, antibiotics on discharge, insurance type	Adjusted RR for 30-day emergency visit (LEP/EP)	LEP associated with increase in ED visits: aRR 2.7 (1.3-5.3); preventable visit, aRR 3.6 (1.7-7.9)	↑

Manuel et al,[27] 2022	30-day readmission after total knee and hip arthroplasty	Multivariable logistic regression model	Age, sex, race/ethnicity, insurance type, ASA status, BMI, surgical case class, case length, and estimated blood loss	Adjusted OR for 30-day readmission (LEP/EP)	No difference in readmission rates: aOR 0.80 (0.49-1.28)	.
Manuel et al,[28] 2022	30-day readmission after craniotomy	Multivariable logistic regression model	Age, sex, race/ethnicity, insurance type, ASA status, and surgical case class	Adjusted OR for 30-day readmission (LEP/EP)	No difference in readmission rates: aOR 0.84 (0.45-1.56)	.
Stolarski et al,[29] 2022	30-day readmission, 1-year readmission, 1-year emergency department visits after laparoscopic sleeve gastrectomy or gastric bypass	Multivariable logistic regression model	Age, sex, race, insurance status, ASA class, smoking status, year of operation, and procedure type	Adjusted OR for 30 day-readmission, 1-year readmission, and 1-year ED revisits, (LEP/EP)	No difference in 30-day readmission, aOR 1.01 (0.58-1.71); No difference in 1-year readmission, aOR 0.94 (0.56-1.55); Fewer 1-year ED revisits in LEP, aOR 0.65 (0.43-0.95)	.↓
**Long-term Mortality or Survival**						
Nashed et al,[15] 2012	Overall survival from time of initial diagnostic surgery to date of death	Log rank test of equality	None	Difference in time-to-event between LEP & EP	No difference in overall survival (p-value = 0.40)	.

© 2023 Joo H et al. *JAMA Network Open.*

Hyun et al,[21] 2017	Death from admission to 18-month follow-up after admission for ACS event	Multivariable logistic regression model	Gender, GRACE risk score, previous cardiac diagnosis and procedures, presenting diagnosis, medications, PCI, CABG, referral to rehabilitation	Adjusted OR for long-term mortality (LEP/EP)	No difference in mortality: aOR 1.08 (0.75-1.58)	.
Sridhar et al,[9] 2019	Overall survival after curative pancreatic cancer surgery	Log rank test of equality	None	Difference in time-to-event between LEP & EP	No difference between LEP and EP in median overall survival from Stage I-II cancer, (p-value=0.778); Stage III-IV cancer, longer median overall survival for LEP than EP (8 vs. 5 months; p-value=0.039)	.↑
Feeney et al,[24] 2020	Time to all-cause mortality from surgical oncology procedure	Multivariable Cox proportional hazard regression model	Age, race/ethnicity, insurance status, risk score, emergency classification, and Elixhauser comorbidity score	Adjusted HR for all-cause mortality (LEP/EP)	No difference in mortality: aHR 0.87 (0.52-1.45)	.
Asokan et al,[10] 2020	Overall survival defined as time from esophageal cancer diagnosis to death censored to last follow-up (in operable patients)	Log rank test of equality	None	Difference in time-to-event between LEP & EP	No difference between LEP and EP in overall survival (p-value=0.718)	.

Abbreviations: AAA, Abdominal aortic aneurysm; ACS, Acute coronary syndrome; aHR, Adjusted hazard ratio; aIRR, Adjusted incidence rate ratio; aMR, Adjusted means ratio; aOR, Adjusted odds ratio; aRR, Adjusted relative risk; ASA, American Society of Anesthesiologists; BMI, Body mass index; CSICU, Cardiovascular surgical intensive care unit; COPD, Chronic obstructive pulmonary disease; CI, Confidence interval; CHF, Congestive heart failure; CABG, Coronary artery bypass surgery; CAD, Coronary artery disease; DM, Diabetes

© 2023 Joo H et al. *JAMA Network Open.*

mellitus; EEG, Electroencephalogram; EPP, Electronic patient portal; ED, Emergency department; EP, English Proficiency; EQ-VAS, EuroQol Group visual analogue scale; EWL, Excess weight loss; GRACE, Global Registry of Acute Coronary Syndrome; HR, Hazard ratio; HTN, Hypertension; IRR, Incidence rate ratio; IKS, International Knee Society; JSN, Joint space narrowing; KL, Kellgren Lawrence classification; LOS, Length of stay; LEP, Limited English proficiency; LEP-I, Limited English proficiency with interpreter required; LEP-N, Limited English proficiency with no interpreter required; MACE, Major adverse cardiovascular events; MIGS, Minimally invasive gynecologic surgery; N/S, non-specified; NSQIP, National Surgical Quality Improvement Program; NENS, Non-English/non-Spanish; OR, Odds ratio; OME, Oral morphine equivalent; OHS, Oxford hip score; PCI, Percutaneous coronary intervention; RR, Relative risk; SES, Socioeconomic status; THA, Total hip arthroplasty; TKA, Total knee arthroplasty.

© 2023 Joo H et al. *JAMA Network Open.*

eTable 2. Decision for Inclusion and Exclusion

Source	Included or Excluded	Reason for Exclusion	Note
**Results of full-text study review**			
Danilowicz et al,[34] 1971	Excluded	Wrong population	The study included pediatric patients.
Dzioba et al,[35] 1984	Excluded	Wrong predictors	LEP was not a prespecified primary/secondary predictor or a part of a prediction set.
Doxey et al,[36] 1988	Excluded	Wrong predictors	LEP was defined as 4-level score
Naylor et al,[37] 1993	Excluded	Wrong predictors	LEP was not a prespecified primary/secondary predictor or a part of a prediction set.
John-Baptiste et al,[38] 2004	Included		
Ernest et al,[39] 2007	Excluded	Wrong outcomes	Primary outcome was cognitive function before surgery.
Bandyopadhyay et al,[40] 2007	Excluded	Wrong predictors	English speaking or non-speaking cultural background was one of the predictors described in the study but not LEP.
Clapp et al, [41] 2007	Excluded	Wrong study design	Qualitative and descriptive study.
Hawley et al,[42] 2008	Excluded	Wrong predictors<br>Wrong outcomes	Race and ethnicity were primary predictors of the study. EP was used to only subcategorize Hispanic population into EP Hispanic and LEP Hispanic group. The study looked at decision making processes
Maly et al,[43] 2009	Excluded	Wrong predictors	LEP was not a prespecified primary/secondary predictor or a part of a prediction set.
Dowsey et al,[44] 2009	Included		
Halpern et al, [45] 2009	Excluded	Conference abstract	
Nielsen et al,[46] 2010	Excluded	Wrong predictors	LEP was not a prespecified primary/secondary predictor or a part of a prediction set. Primary predictors were nativity and race/ethnicity.
MacDonald et al,[5] 2010	Included		

Source	Included or Excluded	Reason for Exclusion	Note
Clark et al,[47] 2011	Excluded	Wrong predictors<br>Wrong outcomes	LEP was not a prespecified primary/secondary predictor or a part of a prediction set. Primary predictor was differential consent tool. Primary outcome was questionnaire on understanding of surgery reflecting efficacy of consent.
Campesino et al,[48] 2012	Excluded	Wrong predictors<br>Wrong outcomes<br>Wrong study design	LEP was not a prespecified primary/secondary predictor or a part of a prediction set. The study looked at decision making processes. Qualitative and descriptive study design with mixed methods.
Nashed et al,[15] 2012	Included		
Alnaes et al,[49] 2012	Excluded	Wrong study design	Qualitative case report.
Betjemann et al, [50] 2013	Included		
Dowsey et al,[51] 2014	Excluded	Wrong predictors	LEP was not a prespecified primary/secondary predictor or a part of a prediction set. Primary predictor was SES, and LEP was just one of the adjusted covariates
Ankuda et al,[52] 2014	Excluded	Wrong predictors<br>Wrong outcomes	LEP was not a prespecified primary/secondary predictor or a part of a prediction set. The study looked at decision making processes and advance directives.
Thompson et al,[16] 2014	Included		
Lopez et al,[53] 2014	Excluded	Wrong outcomes<br>Wrong population	Primary outcome was satisfaction in decision making. Study subjects were recruited from cancer registry, not necessarily a surgical setting.
Alley et al,[54] 2016	Excluded	Wrong predictors	LEP was not a prespecified primary/secondary predictor or a part of a prediction set. Primary predictor was Chinese ethnicity.
Tang et al,[19] 2016	Included		
Patel et al,[55] 2016	Excluded	Wrong predictors	Primary predictor was surgeon's LEP status.

© 2023 Joo H et al. *JAMA Network Open.*

Source	Included or Excluded	Reason for Exclusion	Note
Wilbur et al,[6] 2016	Included		
Inagaki et al,[56] 2016	Excluded	Conference abstract	
Spence et al,[57] 2017	Excluded	Conference abstract	
Talamantes et al,[58] 2017	Excluded	Wrong predictors	LEP was not a prespecified primary/secondary predictor or a part of a prediction set. Primary predictor was zip code with differential proportion of linguistic isolation household.
Inagaki et al, [20] 2017	Included		
Hyun et al,[21] 2017	Included		
Jaiswal et al,[59] 2018	Included		
Yoo et al,[60] 2018	Excluded	Wrong outcomes	Primary outcome was irrigation compliance after surgery
Talutis et al,[61] 2018	Excluded	Conference abstract	
Shiraev et al,[62] 2018	Excluded	Wrong outcomes	Primary outcome was follow-up compliance after surgery
Feeney et al,[23] 2019	Included		
Patel et al,[63] 2019	Excluded	Wrong predictors	LEP was not a prespecified primary/secondary predictor or a part of a prediction set. Primary predictor was hospital type.
Sridhar et al,[9] 2019	Included		
Feeney et al,[64] 2019	Included		
Rosenbloom et al,[65] 2019	Excluded	Wrong predictors	Primary predictor was health care provider's LEP status.
Schultz et al,[66] 2020	Excluded	Conference abstract	
Feeney et al,[24] 2020	Included		
Cataneo et al,[67] 2020	Excluded	Conference abstract	
Asokan et al,[10] 2020	Included		
Bernstein et al,[25] 2020	Included		
Varady et al,[12] 2020	Included		
Hong et al,[68] 2021	Excluded	Conference abstract	
Burgoon et al,[69] 2021	Excluded	Wrong study design	Qualitative and descriptive study.
Wong et al,[33] 2021	Included		
Witt et al,[70] 2021	Included		

Source	Included or Excluded	Reason for Exclusion	Note
Greenberg et al,[71] 2021	Excluded	Wrong predictors	LEP was not a prespecified primary/secondary predictor or a part of a prediction set. Primary predictor was hospital type.
Schwartz et al,[31] 2021	Included		
Witt et al,[13] 2021	Included		
Maurer et al,[14] 2021	Included		
Shehan et al,[72] 2022	Excluded	Wrong outcomes	Primary outcomes were treatment adherence and decision making.
Tang et al,[73] 2022	Excluded	Wrong outcomes	Primary outcome was time to adjuvant chemotherapy. No estimates, 95% CIs, and p-values were reported pertaining to association between LEP and time to surgery.
de Crescenzo et al,[74] 2022	Excluded	Wrong predictors<br>Wrong population	LEP was not a prespecified primary/secondary predictor or a part of a prediction set. Primary predictor was frequency of interpreting service use. Study population consisted of only those who required interpreting service.
Norris et al,[75] 2022	Excluded	Wrong predictors<br>Wrong outcomes	LEP was not a prespecified primary/secondary predictor or a part of a prediction set. Primary outcome was frequency of social needs.
Manuel et al,[28] 2022	Included		
Aggarwal et al,[7] 2022	Included		
Silverstein et al,[8] 2022	Included		
Khan et al,[76] 2022	Excluded	Conference abstract	
Manuel et al,[27] 2022	Included		
Stolarski et al,[29] 2022	Included		
Kovoor et al,[30] 2022	Included		
Dirix et al,[77] 2022	Excluded	Wrong predictors	Primary predicter was language barrier in Belgium
Barnard et al,[78] 2022	Excluded	Wrong predictors	LEP was not a prespecified primary/secondary predictor or a part of a prediction set.

© 2023 Joo H et al. *JAMA Network Open.*

Source	Included or Excluded	Reason for Exclusion	Note
Plocienniczak et al,[79] 2022	Excluded	Wrong population	The study included pediatric patients.
Alwani et al,[80] 2022	Excluded	Wrong outcomes<br>Wrong population	Primary outcome was frequency and length of follow-up care. Study population was not necessarily from a postoperative setting.
**Results of additional studies from relevant systematic reviews**			
Jimenez et al,[81] 2014	Excluded	Wrong population	The study included pediatric patients.
Dai et al,[82] 2021	Excluded	Wrong population	The study included pediatric patients.
Essex et al,[83] 2021	Excluded	Wrong population	The setting was neither surgery nor anesthesiologic procedure
Greene et al,[84] 2019	Excluded	Wrong outcome	Primary outcome was access to initial appointment in orthopedic care, not necessarily implying access to surgical care
Jaramillo et al,[85] 2016	Excluded	Wrong population	The study included pediatric patients.
Lee et al,[86] 2017	Excluded	Wrong predictors	Primary predictor was interpreter intervention among patients with LEP.
Malevanchik et al,[87] 2021	Excluded	Wrong population	The study population was patients discharged from hospital, not necessarily surgical patients.
Plancarte et al,[88] 2021	Excluded	Wrong population	The study included pediatric patients.
Qureshi et al,[89] 2014	Excluded	Wrong population	The study population was patients treated with radiotherapy.

Abbreviations: CI, Confidence interval; EP, English Proficiency; LEP, Limited English proficiency.

© 2023 Joo H et al. *JAMA Network Open.*

eTable 3. Unadjusted Results

Source	Definition of Outcome	Statistical Methodology	Measure of Association	Magnitude and Direction of Associations Between LEP (vs. EP) and Outcome Measures
**Access to Surgery**				
Betjemann et al,[50] 2013	Underwent anterior temporal lobectomy after screening confirmed refractory epilepsy and mesial temporal sclerosis	Pearson's chi-square test	Independence between LEP status and opting into operation	LEP and receipt of surgery were associated (p-value = 0.01)
Sridhar et al,[9] 2019	Receipt of curative pancreatic cancer surgery	Pearson's chi-square test	Independence between LEP status and receipt of surgery	LEP and receipt of surgery were independent (p-value = 0.79)
Asokan et al,[10] 2020	Receipt of esophagectomy in patients with operable stage	Pearson's chi-square test or Fisher's exact test	Independence between LEP status and receipt of surgery	LEP and receipt of surgery were independent (p-value = 0.103)
Witt et al,[70] 2021	Routine admission vs. Emergent/urgent admission for neuro-oncologic surgery	Pearson's chi-square test	Independence between LEP status and rate of emergent/urgent admission	LEP and rate of emergent or urgent admission were associated (p-value < 0.001): The rates were 75.4% for NENS, 61.0% for SPL, and 58.9% for EPL
Maurer et al,[14] 2021	Elective vs. emergent/urgent admission for colectomy	Pearson's chi-square test	Independence between LEP status and rate of emergent/urgent admission	LEP and rate of emergency admission were associated (60.3% vs. 48.6%; p-value < 0.001)

© 2023 Joo H et al. *JAMA Network Open.*

Source	Definition of Outcome	Statistical Methodology	Measure of Association	Magnitude and Direction of Associations Between LEP (vs. EP) and Outcome Measures
**Delay in Surgical Care**				
Nashed et al,[15] 2012	Time from diagnosis to craniotomy	Log rank test of equality	Difference in time-to-event between LEP & EP	No difference in time to surgery (p-value = 0.26).
Thompson et al,[16] 2014	Time from presurgical evaluation to anterior temporal lobectomy	Log rank test of equality	Difference in time-to event between LEP & EP	Patients with LEP had Longer time to surgery than those with EP (p-value = 0.0085)
		Univariate cox proportional hazard regression model	Unadjusted HR for surgery (LEP/EP)	Patients with LEP had longer times to surgery than those with EP: HR 0.56 (95% CI 0.36-0.87)
Jaiswal et al,[59] 2018	Time from breast cancer diagnosis to initial treatment (delayed treatment >37 days; timely treatment <37 days)	Bivariate logistic regression model	Unadjusted OR for delayed treatment (LEP/EP)	LEP associated with longer time to treatment: OR 5.0 (95% CI N/S; p-value=0.0045)
Silverstein et al,[8] 2022	Time from date of referral to first appointment with a MIGS provider (delayed interval > 30 days; timely interval < 30 days)	Pearson's chi-square test or Fisher's exact test	Independence between LEP status and timely interval for appointment	LEP and timely interval were associated in the pandemic cohort (p-value < 0.01): The rates of having timely interval were 45.7%% for LEP and 71.6% for EP; LEP and timely interval were independent in the historic cohort (p-value = 0.84): The rates of having timely interval were 38.7%% for LEP and 40.5% for EP.
**Length of Stay**				
MacDonald et al,[5] 2010	LOS (long LOS, 7>days; short LOS, 6<days) after hip and knee arthroplasty	Pearson's chi-square test	Independence between LEP status and long LOS	LEP and proportion of long LOS were associated (p-value < 0.05).

© 2023 Joo H et al. *JAMA Network Open.*

Source	Definition of Outcome	Statistical Methodology	Measure of Association	Magnitude and Direction of Associations Between LEP (vs. EP) and Outcome Measures
Tang et al,[19] 2016	LOS defined as time from CSICU admission to hospital discharge after CABG. LOS was categorized into quartiles (1-5 days, 6 days, 7-8 days, and >9 days)	Multivariate polynomial regression model	Unadjusted OR for each quartile of LOS (LEP/EP)	Increased or no difference in LOS: LOS 6 days vs LOS 1-5 days OR 1.78 (0.96-3.29); LOS 7-8 days vs. LOS 1-5 days OR 1.59 (0.87-2.91); LOS ≥9 days vs. 1-5 days OR 2.17 (1.21-3.91).
Inagaki et al,[20] 2017	Postoperative LOS after infrainguinal bypass surgery	Unpaired t-test	Difference in mean LOS between LEP & EP	No difference in LOS between LEP and EP (11.2 days vs. 9.4 days; p-value = 0.202).
Hyun et al,[21] 2017	LOS in days after admission with suspected ACS event	Wilcoxon rank-sum test	Difference in median LOS between LEP & EP	Longer LOS for those with LEP than EP (3.1 vs. 2.5 days; p-value = 0.001).
Feeney et al,[64] 2019	LOS in days after oncologic surgery	Generalized linear model with negative binomial distribution	Unadjusted IRR for LOS (Spanish/EP OR NENS/EP)	Increased LOS for NENS: LEP (Spanish) vs. EP, IRR 1.04 (0.99-1.10); LEP (NENS) vs. EP, IRR 1.06 (1.01-1.12).
Feeney et al,[23] 2019	LOS during hospitalization for emergency surgery	Generalized linear model with negative binomial distribution	Unadjusted IRR for LOS (Spanish/EP OR NENS/EP)	Spanish speakers had reduced LOS: LEP (Spanish) vs. EP, IRR 0.72 (0.71-0.74), LEP (NENS) vs. EP, IRR 0.96 (0.93-0.98)
Feeney et al,[24] 2020	LOS in days	Kruskal Wallis rank-sum test	Difference in median LOS between LEP & EP	No difference in median LOS between LEP and EP (1.5 vs. 1.5; p-value = 0.68).
Witt et al,[70] 2021	Total LOS after neuro-oncologic surgery	Bivariate negative binomial regression model	Unadjusted IRR for LOS (Spanish/EP OR NENS/EP)	Increased LOS for NENS: LEP (Spanish) vs. EP, IRR 1.05 (0.97-1.13); LEP (NENS) vs. EP, IRR 1.29 (1.19-1.39)
	Postoperative LOS after neuro-oncologic surgery		Unadjusted IRR for postoperative LOS (Spanish/EP OR NENS/EP)	Increased postop LOS for NENS: LEP (Spanish) vs. EP, IRR 1.01 (0.93-1.09); LEP (NENS) vs. EP, IRR 1.32 (1.23-1.44)
Manuel et al,[27] 2022	Total LOS after total knee and hip arthroplasty	Wilcoxon rank-sum test	Difference in median LOS between LEP & EP	Longer median LOS in patient with LEP than EP (p-value < 0.001)

© 2023 Joo H et al. *JAMA Network Open.*

Source	Definition of Outcome	Statistical Methodology	Measure of Association	Magnitude and Direction of Associations Between LEP (vs. EP) and Outcome Measures
Manuel et al,[28] 2022	Total LOS after craniotomy	Bivariate negative binomial regression model	Unadjusted IRR for LOS (LEP/EP)	Longer LOS for LEP: IRR 1.41 (1.26-1.58)
Stolarski et al,[29] 2022	LOS from index operation (laparoscopic sleeve gastrectomy or gastric bypass)	Wilcoxon rank-sum test	Difference in median LOS between LEP & EP	No difference in median LOS between LEP and EP (2.26 days vs. 2.12 days; p-value = 0.60).
**Discharge Disposition**				
Witt et al,[70] 2021	Discharge disposition to rehabilitation (vs. home) after neuro-oncologic surgery	Pearson's chi-square test	Independence between LEP status and discharge disposition to home	LEP and proportion of discharge disposition to home were associated (p-value < 0.001): The proportions were 72.4% for LEP (Spanish), 57.6% for NENS, and 60.0% for EP.
Manuel et al,[27] 2022	Discharge disposition to skilled facility (vs. home) after total knee or hip arthroplasty	Pearson's chi-square test	Independence between LEP status and discharge disposition to skilled facility	LEP and discharge dispositions to skilled facility were associated (42.6% vs. 20.5%; p-value < 0.001)
Manuel et al,[28] 2022	Discharge disposition to skilled facility (vs. home) after craniotomy	Bivariate logistic regression model	Unadjusted OR for skilled facility discharge (LEP/EP)	Increased discharge to skilled facility for LEP: OR 2.26 (1.60-3.20)
**In-hospital Mortality**				
Hyun et al,[21] 2017	In-hospital death during admission for suspected ACS event	Pearson's chi-square test	Independence between LEP status and in-hospital death	LEP and in-hospital mortality were associated (4.4% vs. 1.7%; p-value = 0.001).
Feeney et al,[64] 2019	In-hospital all-cause mortality after oncologic surgery	Generalized linear model with Bernoulli distribution	Unadjusted OR for in-hospital death (Spanish/EP OR NENS/EP)	No difference in mortality: LEP (Spanish) vs. EP, OR 0.73 (0.49-1.08); LEP (NENS) vs. EP, OR 1.30 (0.94-1.76).

© 2023 Joo H et al. *JAMA Network Open.*

Source	Definition of Outcome	Statistical Methodology	Measure of Association	Magnitude and Direction of Associations Between LEP (vs. EP) and Outcome Measures
Feeney et al,[23] 2019	In-hospital all-cause mortality after emergency surgery	Generalized linear model with Bernoulli distribution	Unadjusted OR for in-hospital death (Spanish/EP OR NENS/EP)	Reduced or no difference in LOS: LEP (Spanish) vs. EP, OR 0.33 (0.26-0.43), LEP (NENS) vs. EP, OR 0.92 (0.74-1.13).
Witt et al,[70] 2021	In-hospital mortality after neuro-oncologic surgery	Pearson's chi-square test	Difference in in-hospital mortality across LEP statuses	No difference across LEP statuses in in-hospital mortality (p-value =0.127)
**Complication**				
Inagaki et al,[20] 2017	30-day would infections, 30-day adverse graft event after infrainguinal bypass surgery	Pearson's chi-square test	Independence between LEP status and 30-day would infections	LEP and wound infections are independent (31.4% vs. 35.7%; p-value = 0.415)
			Independence between LEP status and 30-day adverse graft events	LEP and adverse graft events were independent (31.4% vs. 29.0%; p-value = 0.744)
Hyun et al,[21] 2017	In-hospital MACE; Delayed <18 months MACE after admission for ACS event	Pearson's chi-square test	Independence between LEP status and in-hospital MACE	LEP and in-hospital MACE were associated (24.2% vs. 14.9%; p-value < 0.001)
			Independence between LEP status and 18-month MACE	LEP and 18-month MACE were independent (22.5% vs. 20.7%; p-value = 0.59)
Feeney et al,[24] 2020	In-hospital major complications after cancer surgery based on NSQIP risk calculator major morbidity definition	Pearson's chi-square test	Independence between LEP status and in-hospital major complications	LEP and major complications were independent (2.3% vs. 3.3%; p-value = 0.19).

© 2023 Joo H et al. *JAMA Network Open.*

Source	Definition of Outcome	Statistical Methodology	Measure of Association	Magnitude and Direction of Associations Between LEP (vs. EP) and Outcome Measures
Witt et al,[70] 2021	Presence of inpatient complications validated for supratentorial tumors	Pearson's chi-square test	Independence between LEP status and inpatient complications	LEP and proportion of inpatient complications were associated (p-value < 0.001): The proportions were 16.2% for LEP (Spanish), 28.8% for NENS, and 19.0% for EP.
Stolarski et al,[29] 2022	Presence of 30-day complications after laparoscopic sleeve gastrectomy or gastric bypass	Pearson's chi-square test	Independence between LEP status and 30-day complications	LEP and 30-day complications were independent (2.0% vs. 1.0%; p-value = 0.08)
**Pain Management**				
Schwartz, et al,[31] 2021	Total oral morphine equivalent (OME) prescribed at discharge after admission to trauma surgery service	Bivariate logistic regression model	Unadjusted OR for receiving discharge opioids (LEP/EP)	LEP less likely to receive discharge opioid prescription: OR 0.56 (0.42-0.75)
	Total amount of opioids prescribed at discharge after admission to trauma surgery service	Bivariate quantile regression model	Difference in mean total OME between LEP & EP	LEP patients received 75.0 (43.5-106.6) fewer OME than EP patients at 60th percentile; LEP patients received 75.0 (33.8-116.2) fewer OME than EP patients at 80th percentile
**Long-term Outcome**				
Dowsey, et al,[44] 2009	International Knee Society (IKS) score measure of function 12 months after TKA	Pearson's chi-square test	Independence between LEP status and having poor grade (≤120) 12-month IKS score	Right TKA, LEP and poor grade IKS score were associated (58% vs. 27%; p-value < 0.001).

© 2023 Joo H et al. *JAMA Network Open.*

Source	Definition of Outcome	Statistical Methodology	Measure of Association	Magnitude and Direction of Associations Between LEP (vs. EP) and Outcome Measures
**Readmission**				
Inagaki et al,[20] 2017	Unplanned readmission within 30-days of discharge, emergency department (ED) visit within 30 days of discharge after infrainguinal bypass surgery	Pearson's chi-square test	Independence between LEP status and 30-day readmissions<br><br>Independence between LEP status and 30-day ED visits	LEP and 30-day readmissions were independent (25.5% vs. 20.4%; p-value = 0.426).<br><br>LEP and 30-day ED visits were independent (23.5% vs. 27.1%; p-value = 0.600).
Feeney et al,[64] 2019	7-day readmission after discharge from oncologic surgery	Generalized linear model with Bernoulli distribution	Unadjusted OR for 7-day readmission (Spanish/EP OR NENS/EP)	LEP associated with higher readmission rates: LEP (Spanish) vs. EP, 1.35 (1.04-1.75); LEP (NENS) vs. EP, 1.50 (1.11-2.02).
Feeney et al,[23] 2019	7-day readmission after discharge from emergency surgery admission	Generalized linear model with Bernoulli distribution	Unadjusted OR for 7-day readmission (Spanish/EP OR NENS/EP)	LEP associated with lower readmission rates: LEP (Spanish) vs. EP, OR 0.70 (0.57-0.84), LEP (NENS) vs. EP, OR 0.78 (0.60-0.98).
Feeney et al,[24] 2020	30-day revisit to emergency department after discharge from cancer surgery	Pearson's chi-square test	Independence between LEP status and 30-day ED visits	LEP and 30-day ED visits were independent (12.0% vs. 11.0%; p-value = 0.50).
Wong et al,[33] 2021	Postoperative emergency department visit within 30 days of discharge	Pearson's chi-square test	Independence across LEP status, preventable 30-day ED visits, and nonpreventable 30-day ED visits	LEP and either preventable or nonpreventable ED visits were associated (p-value < 0.001).
Manuel et al,[27] 2022	30-day readmission after total knee and hip arthroplasty	Pearson's chi-square test	Independence between LEP status and 30-day readmissions	LEP and 30-day readmissions were independent (7.9% vs. 7.2%; p-value = 0.59).
Manuel et al,[28] 2022	30-day readmission after craniotomy	Bivariate logistic regression model	Unadjusted OR for 30-day readmission (LEP/EP)	No difference in readmission rates: OR 1.26 (0.73-2.17).

© 2023 Joo H et al. *JAMA Network Open.*

Source	Definition of Outcome	Statistical Methodology	Measure of Association	Magnitude and Direction of Associations Between LEP (vs. EP) and Outcome Measures
Stolarski et al,[29] 2022	30-day readmission, 1-year readmission, 1-year emergency department visits after laparoscopic sleeve gastrectomy or gastric bypass	Pearson's chi-square test	Independence between LEP status and 30-day readmissions	LEP and 30-day readmissions were independent (4.0% vs. 5.0%; p-value = 0.70).
			Independence between LEP status and 1-year readmissions	LEP and 1-year readmissions were independent (8.0% vs. 10.0%; p-value = 0.20) .
			Independence between LEP status and 1-year ED visits	LEP and 1-year ED visits were associated (14.0% vs. 23.0%; p-value < 0.001) .
**Long-term Mortality or Survival**				
Nashed et al,[15] 2012	Overall survival from time of initial diagnostic surgery to date of death	Log rank test of equality	Difference in time-to-event between LEP & EP	No difference between LEP and EP in overall survival (p-value = 0.40)
Hyun et al,[21] 2017	Death from admission to 18-month follow-up after admission for ACS event	Pearson's chi-square test	Independence between LEP status and 18-month mortality	LEP and 19-month mortality were associated (16.3% vs. 10.1%; p-value = 0.001)
Sridhar et al,[9] 2019	Overall survival after curative pancreatic cancer surgery	Log rank test of equality	Difference in time-to-event between LEP & EP	No difference between LEP and EP in median overall survival from Stage I-II cancer (p-value = 0.778); Stage III-IV cancer, longer median overall survival for LEP than EP (8 vs. 5 months; p-value = 0.039)
Feeney et al,[24] 2020	Time to all-cause mortality from surgical oncology procedure	Log rank test of equality	Difference in time-to-event between LEP & EP	Longer time to death (survival) for LEP than EP (p-value = 0.00063).

© 2023 Joo H et al. *JAMA Network Open.*

Source	Definition of Outcome	Statistical Methodology	Measure of Association	Magnitude and Direction of Associations Between LEP (vs. EP) and Outcome Measures
Asokan et al,[10] 2020	Overall survival defined as time from esophageal cancer diagnosis to death censored to last follow-up (in operable patients)	Log rank test of equality	Difference in time-to-event between LEP & EP	No difference between LEP and EP in overall survival (p-value = 0.718)

Abbreviations: ACS, Acute coronary syndrome; CSICU, Cardiovascular surgical intensive care unit; CI, Confidence interval; CABG, Coronary artery bypass surgery; ED, Emergency department; EP, English Proficiency; HR, Hazard ratio; IRR, Incidence rate ratio; IKS, International Knee Society; LOS, Length of stay; LEP, Limited English proficiency; MACE, Major adverse cardiovascular events; MIGS, Minimally invasive gynecologic surgery; N/S, non-specified; NSQIP, National Surgical Quality Improvement Program; NENS, Non-English/non-Spanish; OR, Odds ratio; OME, Oral morphine equivalent; TKA, Total knee arthroplasty.

© 2023 Joo H et al. *JAMA Network Open.*

Supplementary Figures

eFigure 1. Articles per Year that met Inclusion Criteria

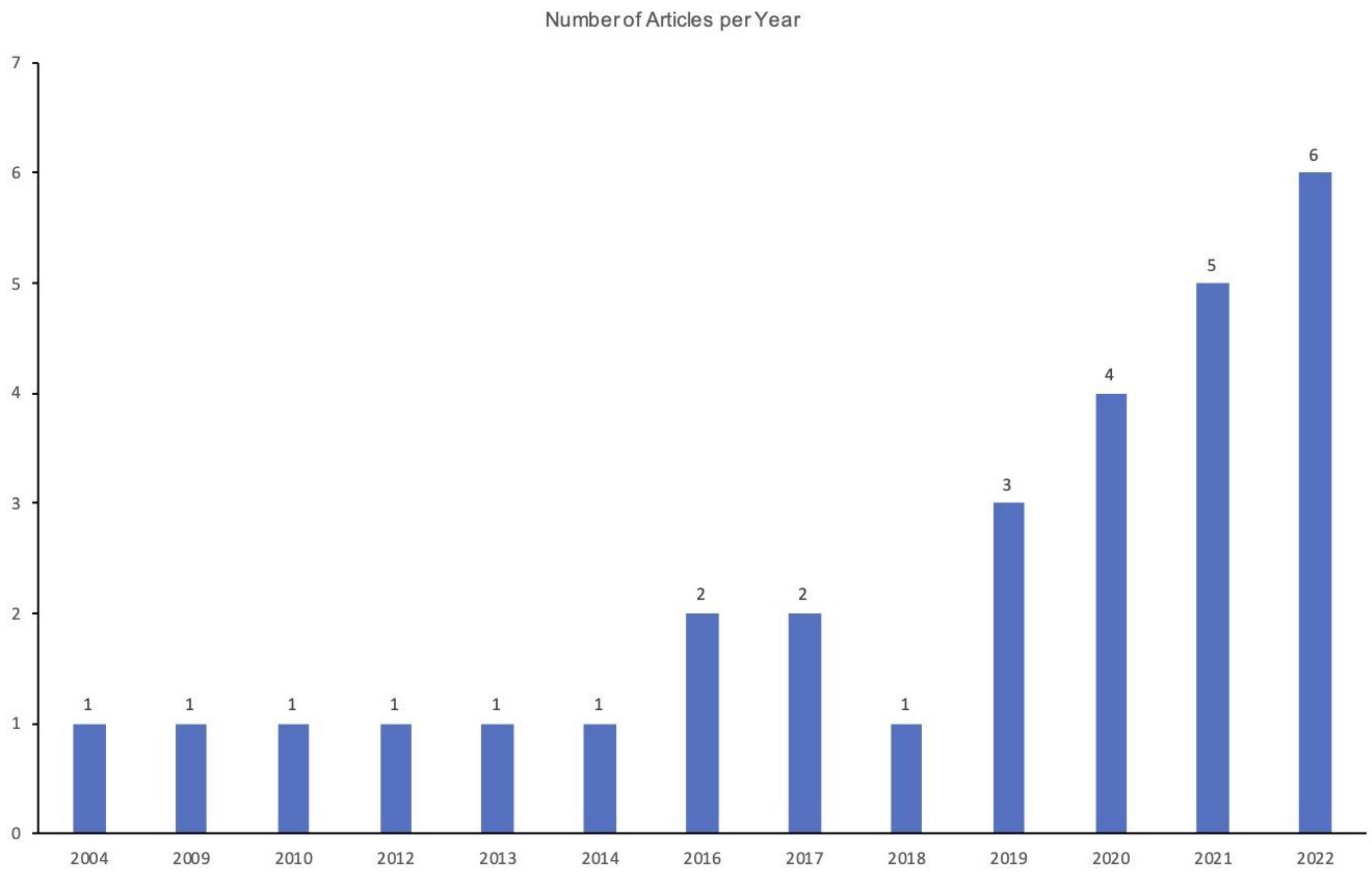

© 2023 Joo H et al. *JAMA Network Open.*

eFigure 2. Perioperative Outcomes Represented

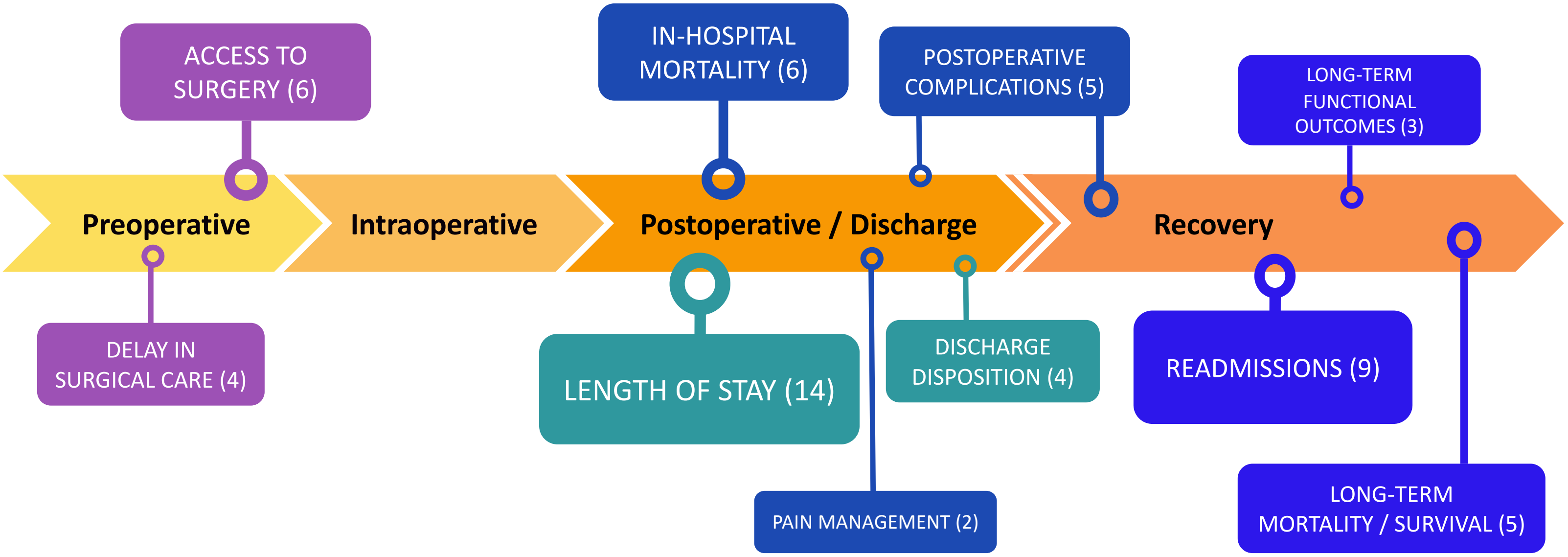

© 2023 Joo H et al. *JAMA Network Open.*
