# Supplementary material for: Association of Language Barriers With Perioperative and Surgical Outcomes: A Systematic Review
Source: JAMA Netw Open. 2023 Jul 11;6(7):e2322743. doi: 10.1001/jamanetworkopen.2023.22743 (PMC10336626; doi:10.1001/jamanetworkopen.2023.22743)
Supplement: Supplement 2. — Data Sharing Statement [file jamanetwopen-e2322743-s002.pdf]

Data Sharing Statement

Joo. Association of Language Barriers With Perioperative and Surgical Outcomes. *JAMA Netw Open*. Published July 11, 2023. doi:10.1001/jamanetworkopen.2023.22743

Data
Data available: Yes
Data types: Data (not involving human participants)
How to access data: The complete study search strategy and search terms, along with the full list of articles that underwent full text review will be available in the eSupplement of this manuscript.
When available: With publication

Supporting Documents
Document types: None

Additional Information
Who can access the data: Additional data and analytic code pertaining to this study will be made available without restriction to anyone upon request.
Types of analyses: No restriction.
Mechanisms of data availability: The complete study search strategy and search terms, along with the full list of articles that underwent full text review will be available in the eSupplement of this manuscript. Any additional data and analytic code pertaining to this study will be made available without restriction to anyone upon request.
